# A Synergistic Dual‐Atom Sites Nanozyme Augments Immunogenic Cell Death for Efficient Immunotherapy

**DOI:** 10.1002/advs.202414734

**Published:** 2024-12-24

**Authors:** Shipeng Ning, Zeyuan Zhang, Yujing Ren, Yaxin Hou, Dan Li, Jingqi Chen, Yujie Zhai, Kelong Fan, Weiqing Zhang

**Affiliations:** ^1^ Department of Breast Surgery The Second Affiliated Hospital of Guangxi Medical University Nanning 530000 China; ^2^ Department of Research Guangxi Medical University Cancer Hospital Guangxi Medical University Nanning 530021 China; ^3^ University Engineering Research Center of Oncolytic & Nanosystem Development Guangxi 530021 China; ^4^ West China School of Medicine Sichuan University Chengdu 610041 China; ^5^ Interdisciplinary Research Center of Biology & Catalysis School of Life Sciences Northwestern Polytechnical University Xi'an 710072 China; ^6^ CAS Engineering Laboratory for Nanozyme Key Laboratory of Biomacromolecules (CAS) CAS Center for Excellence in Biomacromolecules Institute of Biophysics Chinese Academy of Sciences Beijing 100101 China; ^7^ Nanozyme Laboratory in Zhongyuan Henan Academy of Innovations in Medical Science Zhengzhou Henan 451163 China

**Keywords:** cancer immunotherapy, cascaded catalysis, dual‐atom nanozyme, immunogenic cell death, photothermal therapy

## Abstract

Inducing immunogenic cell death (ICD) is a promising approach to elicit enduring antitumor immune responses. Hence, extensive efforts are being made to develop ICD inducers. Herein, a cascaded dual‐atom nanozyme with Fe and Cu sites (FeCu‐DA) as an efficient ICD inducer is presented. The Fe and Cu dual‐atom sites synergistically enhance peroxidase (POD) and catalase activities, effectively converting intratumoral hydrogen peroxide (H_2_O_2_) to hydroxyl radicals (·OH) and oxygen (O_2_). Moreover, FeCu‐DA exhibits superior glutathione‐oxidase (GSH‐OXD) activity, catalyzing GSH oxidation to generate H_2_O_2_, enabling cascaded catalysis for sustainable ∙OH generation and reducing reactive oxygen species (ROS) resistance by consuming GSH. Steady–state kinetic analysis and density functional theory calculations indicate that FeCu‐DA exhibits a higher catalytic rate and efficiency than Fe single‐atom nanozymes (Fe‐SA) because of its stronger interactions with H_2_O_2_. Its POD activity is 948.05 U mg^−1^, which is 2.8‐fold greater than that of Fe‐SA. Furthermore, FeCu‐DA exhibits impressive photothermal effects and photothermal‐enhanced cascaded catalysis kinetics for ROS generation, thereby inducing potent ICD. Combined with anti‐PD‐L1 antibody (αPD‐L1) blockade, FeCu‐DA shows synergistic enhancement in treatment under near‐infrared irradiation. This study provides insights for designing efficient dual‐atom nanozymes and demonstrates their potential in ICD‐induced cancer immunotherapy.

## Graphical abstract

The dual‐atom nanozymes with Fe and Cu sites demonstrate remarkable photothermal effects and significantly enhanced cascaded catalytic activity, enabling sustained reactive oxygen species generation. This dramatically enhances the therapeutic efficacy of cancer immunotherapy by inducing robust immunogenic cell death (ICD). The study provides valuable insights for designing efficient dual‐atom nanozymes and highlights their promising potential in ICD‐mediated cancer immunotherapy.

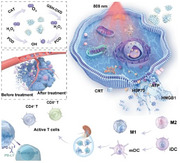

## 1 Introduction

Immunotherapy, a cutting‐edge cancer treatment, activates intratumoral T cells to trigger an immune response that eliminates tumor cells.^[^ ^1^ ^]^ However, its tremendous potential is currently limited by the highly immunosuppressive tumor microenvironment (TME) and insufficient tumor immunogenicity.^[^ ^2^ ^]^ Immunogenic cell death (ICD) is a form of cell death characterized by the release of tumor‐associated antigens, damage‐associated molecular patterns (DAMPs), and proinflammatory cytokines.^[^ ^3^ ^]^ These released molecules play pivotal roles in activating T cells and enhancing the immune microenvironment.^[^ ^4^ ^]^ Significant efforts are underway to develop ICD inducers for immunotherapy. For instance, chemotherapy‐based ICD inducers (such as oxaliplatin) have shown ICD‐inducing effects at high concentrations, which can cause considerable toxicity to healthy organs.^[^ ^5^ ^]^ There is a pressing need to develop efficient ICD inducers capable of stimulating a robust immune response, which could significantly increase the efficacy of cancer immunotherapy.

The induction of ICD is closely associated with the generation of reactive oxygen species (ROS).^[^ ^6^ ^]^ Peroxidase (POD) nanozymes are particularly effective at inducing ICD by converting excessive intracellular hydrogen peroxide (H_2_O_2_) into highly cytotoxic hydroxyl radicals (·OH).^[^ ^7^ ^]^ Recently, single‐atom nanozymes (SAzymes) with metal‐N_x_‐C configurations have garnered increasing attention in the fields of ICD induction and immunotherapy.^[^ ^8^, ^9^ ^]^ These SAzymes have multiple enzymatic activities, including POD, catalase (CAT) and glutathione‐oxidase (GSH‐OXD),^[^ ^10^ ^]^ making them highly effective at increasing ROS levels by initiating cascaded catalytic reactions within the intricate TME. In addition, GSH depletion within the TME disrupts the antioxidant defense system, preventing ROS neutralization and further amplifying oxidative stress.^[^ ^11^ ^]^ Despite these advantages, the enzymatic performance of SAzymes still remains insufficient to fully meet therapeutic demands. Continued innovations are needed to enhance their catalytic efficiency and overall efficacy in inducing robust ICD, thereby improving the therapeutic outcomes of cancer immunotherapy.

**Scheme 1:**
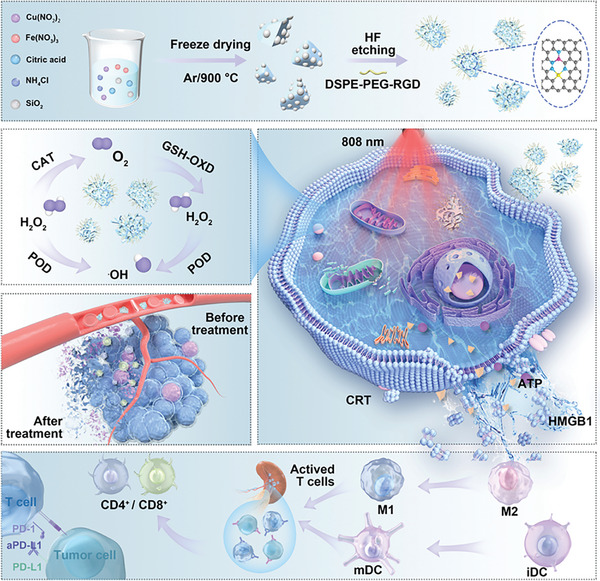
Schematic illustration of the synthetic process and therapeutic mechanism of FeCu‐DA.

The incorporation of a second metal into metal‐N_x_‐C configurations is an efficient strategy for enhancing their catalytic efficiency.^[^ ^12^ ^]^ The electron orbital hybridization of dual metal atoms allows for better tunability of their electron structures and enzymatic activities.^[^ ^13^ ^]^ In this study, we successfully synthesize a cascaded dual‐atom nanozyme with Fe and Cu sites (FeCu‐DA) for ICD induction and immunotherapy. FeCu‐DA exhibits superior cascaded catalytic activities, mimicking the functions of POD, CAT and GSH‐OXD, significantly outperforming Fe single‐atom nanozymes (Fe‐SA) (**Scheme** **1**). Specifically, FeCu‐DA effectively converts intratumor H_2_O_2_ to ∙OH and oxygen (O_2_) because of its excellent POD and CAT activities. In the presence of O_2_, FeCu‐DA also catalyzes the oxidation of GSH to generate H_2_O_2_, which is subsequently converted to ∙OH and O_2_. This sustainable generation of ∙OH, coupled with GSH depletion, amplifies oxidative stress and exacerbates the redox imbalance. Furthermore, FeCu‐DA shows excellent photothermal effects, further enhancing its cascaded catalytic activities under near‐infrared (NIR) irradiation. Even at a low loading dose of 20 µg mL^−1^, FeCu‐DA effectively induces robust ICD, leading to significant immune responses and therapeutic efficacy against breast cancer (Scheme 1). Moreover, when combined with anti‐PD‐L1 antibody (αPD‐L1) immunotherapy, FeCu‐DA synergistically enhances treatment outcomes, offering a promising strategy for advancing cancer therapy.

## 2 Results and Discussion

### 2.1 Synthesis and Characterization of FeCu‐DA

**Figure 1:**
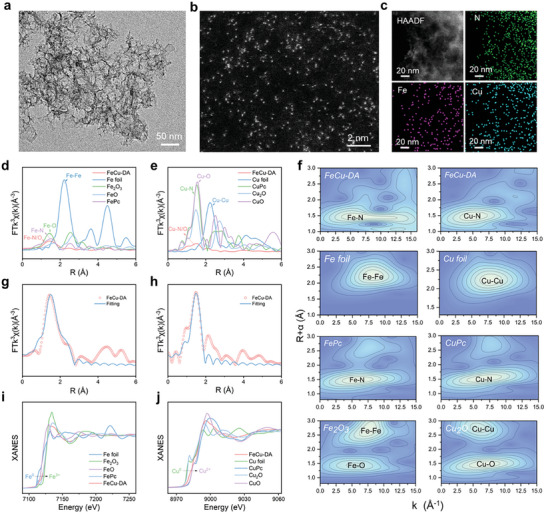
Characterization of FeCu‐DA. a) TEM images. b) HAADF‐STEM images. c) Corresponding EDS mappings. d,e) Fe and Cu K‐edges of Fourier transformed k^3^‐weighted EXAFS spectra of FeCu‐DA and reference samples. f) Wavelet transforms of the Fe and Cu K‐edges in FeCu‐DA and reference samples. g,h) Fitting results of FT‐EXAFS for FeCu‐DA in the Fe‐R and Cu‐R spaces. i,j) Fe and Cu K‐edge XANES spectra.

The FeCu‐DA was synthesized via a pyrolysis method with sacrificial SiO_2_ nanospheres as templates. The solid blend was obtained by lyophilizing an aqueous solution containing SiO_2_ nanospheres, citric acid, NH_4_Cl, Fe(NO_3_)_3_⋅9H_2_O, and Cu(NO_3_)_2_. In this mixture, citric acid chelates with Fe^3+^ and Cu^2+^ ions to form Fe─O and Cu─O chelation moieties. When the solid blend was subjected to pyrolysis at 900 °C in an Ar atmosphere, citric acid and NH_4_Cl were carbonized, resulting in the formation of N‐doped carbon on the surface of the SiO_2_ template. Simultaneously, the Fe─O and Cu─O moieties were transformed into N‐coordinated Fe and Cu atomic sites. N and O are more electronegative than C, which is beneficial for anchoring single metal atoms. After etching the SiO_2_ template with hydrofluoric acid (HF), a nanoporous hierarchical structure of FeCu‐DA was successfully obtained. As a control sample, Fe‐SA was prepared by separately introducing Fe(NO_3_)_3_⋅9H_2_O via the same procedure except for the addition of Cu(NO_3_)_2_ (Figure S1, Supporting Information). The nanoporous architecture has a high surface area, which promotes the exposure of more active sites.^[^ ^14^ ^]^ The hydrodynamic size of FeCu‐DA was determined to be ≈300 nm (Figures S2 and S3, Supporting Information). As shown in **Figure** **1**a, FeCu‐DA possesses a nanoporous hierarchical structure. No metallic or oxidic phases of the Fe or Cu nanoparticles are observed on the carbon support. X‐ray powder diffraction (XRD) patterns of FeCu‐DA exclusively exhibited broad carbon peaks, indicating an amorphous carbon structure. No peaks corresponding to metallic Fe, Cu or other compounds were detected (Figure S4, Supporting Information). High‐angle annular dark‐field scanning TEM (HAADF‐STEM) further revealed a uniform distribution of bright white spots on the porous framework (Figure 1b), indicating the presence of individual Fe and Cu metal atoms rather than clusters or nanoparticles. The Fe and Cu contents were determined via inductively coupled plasma‒optical emission spectrometry (ICP‒OES) to be 0.8 and 0.4 wt%, respectively. Energy dispersive X‐ray spectroscopy (EDS) mapping revealed a uniform dispersion of Fe and Cu atoms (Figure 1c). The Raman spectrum of FeCu‐DA revealed two graphitic D and G bands with a low I_D_/I_G_ ratio (Figure S5, Supporting Information), suggesting the formation of an amorphous carbon structure. This could be attributed to the distortion of the planar structure caused by the Fe and Cu atoms within the graphitic layer, resulting in increased amorphousness.^[^ ^15^ ^]^

Fourier transform extended X‐ray absorption fine structure (EXAFS) spectroscopy was employed to investigate the coordination environment of the Fe and Cu sites in FeCu‐DA. As shown in Figure 1d,e, the Fe and Cu K‐edge EXAFS spectra of FeCu‐DA exhibited a prominent peak at ≈1.44 and 1.50 Å, indicating the coordination of Fe and Cu with light elements such as C, N, and O.^[^ ^16^ ^]^ The absence of peaks at 2.21 and 2.24 Å suggested a lack of coordination between Fe‒Fe, Cu‒Cu, and Fe‒Cu in FeCu‐DA, potentially attributed to the dispersion of individual Fe and Cu atoms. The N 1 s XPS spectra revealed evident Metal‒N bonds in FeCu‒DA, whereas no M─O bonds were observed in the O 1 s XPS spectra (Figure S6, Supporting Information). The wavelet transforms of EXAFS exhibited a singular peak intensity at ≈6.00 and 5.10 Å^−1^, in contrast to the observed behavior in the reference samples (Figure 1f). This observation also substantiated the dispersion of Fe and Cu as individual atoms in FeCu‐DA. EXAFS fitting results further explored the coordination number (CN) of Fe and Cu single atoms in FeCu‐DA (Figure 1g,h; Table S1, Supporting Information). In detail, FeCu‐DA showed Fe─N/C coordination at a distance of 2.00 Å with a CN of 3.8, accompanied by another Fe─N/C coordination at 2.58 Å with a CN of 2.0. The appearance of Fe─N/C coordination at 2.58 Å may be attributed to the amorphous nature of the carbon support.^[^ ^15^ ^]^ The CN of the Cu─N/C coordination at ≈2.0 Å was determined to be 2.9. The aforementioned structural analysis confirmed the presence of single‐atom Fe and Cu in FeCu‐DA with the coordination of Fe─N/C and Cu─N/C. The electronic structures of the Fe and Cu atoms in FeCu‐DA were investigated through X‐ray absorption near‐edge structure (XANES) spectroscopy. As revealed from the Fe K‐edge XANES spectra in Figure 1i, the edge position of Fe atoms was located between Fe foils and Fe_2_O_3_, indicating the oxidation state of the Fe in FeCu‐DA. Similarly, the Cu K‐edge XANES spectra revealed a positive valence state for the Cu atom ranging from +1 to +2, as evidenced by its edge position situated between Cu_2_O and CuO (Figure 1j).

### 2.2 Enzymatic Activities and Photothermal Efficacy Conversion of FeCu‐DA

**Figure 2:**
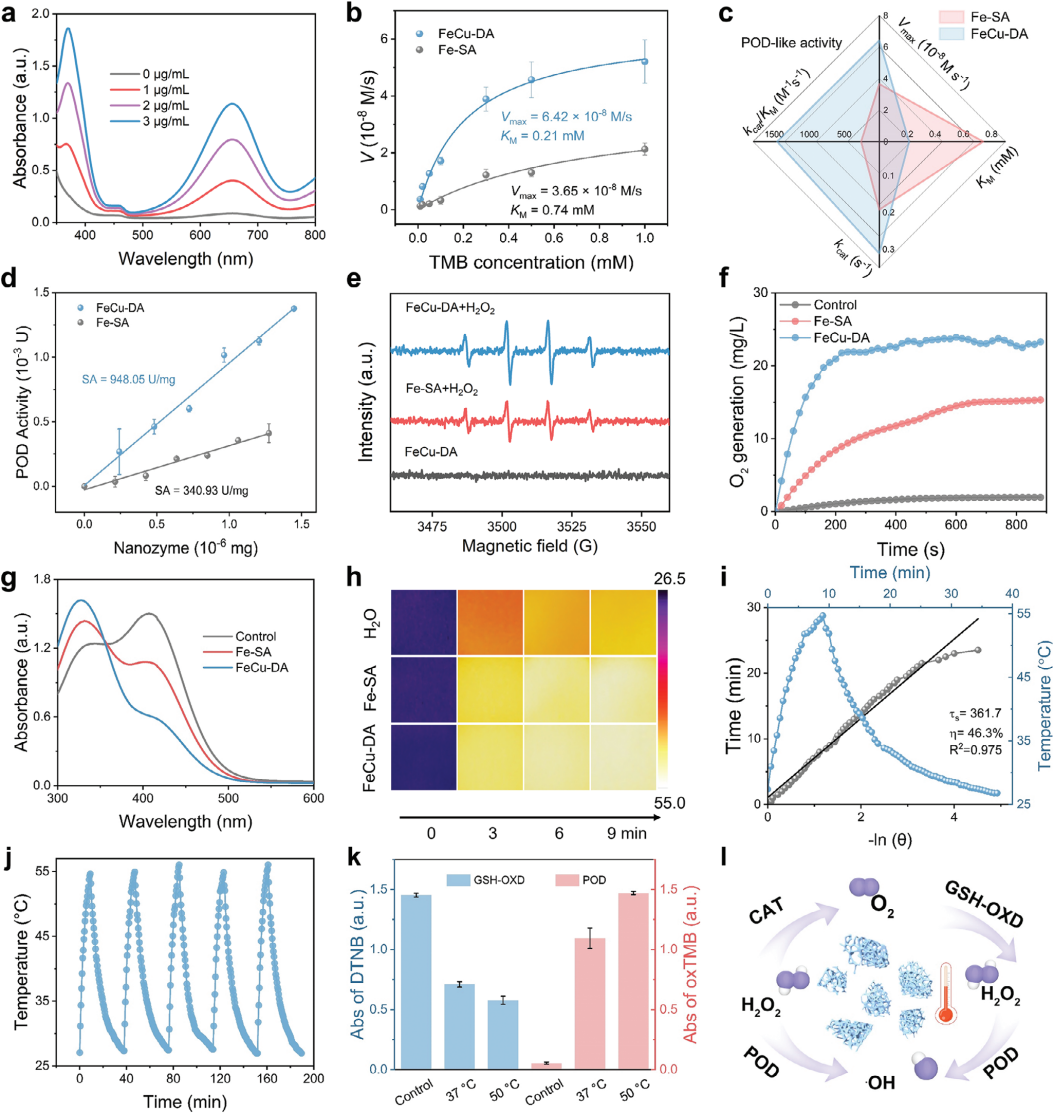
Catalytic performance and photothermal properties of FeCu‐DA. a) POD activity of FeCu‐DA at different concentrations. b) Michaelis‒Menten curves for FeCu‐DA; TMB is the substrate. c) Kinetics parameters of FeCu‐DA and Fe‐SA; TMB is the substrate. d) POD specific activities of FeCu‐DA and Fe‐SA. e) ESR spectra of DMPO‐OH of FeCu‐DA and Fe‐SA. f) CAT activity of FeCu‐DA and Fe‐SA. g) GSH‐OXD activity of FeCu‐DA and Fe‐SA. h) Thermal images under NIR irradiation. i) Photothermal conversion efficiency (*η*) of FeCu‐DA. j) Temperature variation over five on/off cycles of FeCu‐DA under 808 nm laser irradiation. k) Comparison of POD and GSH‐OXD activities at different temperatures. l) Scheme of the PTT‐enhanced cascaded catalytic mechanism of FeCu‐DA. All the quantitative data are presented as the means ± SDs (*n* = 3).

The ∙OH generation can be confirmed via a colorimetric method with 3,3′,5,5′‐tetramethylbenzidine (TMB) as a chromogenic substrate. When exposed to ·OH, TMB undergoes oxidation and transforms into an oxidized compound (ox‐TMB), which exhibits a prominent absorption peak at 652 nm. As shown in **Figure** **2**a, the absorbance of ox‐TMB increased proportionally with the concentration of FeCu‐DA. Furthermore, compared with Fe‐SA, FeCu‐DA significantly enhanced the characteristic absorbance of ox‐TMB, demonstrating its superior POD activity (Figure S7, Supporting Information). The POD catalytic performance of FeCu‐DA was further evaluated through steady‐state kinetic analysis. Key kinetic parameters, including the Michaelis constant (*K*_M_) and maximum velocity (*V*_max_), were determined for FeCu‐DA and Fe‐SA (Figure 2b). The *K*_M_ values of FeCu‐DA are 3.8 and 3.5 times lower than those of Fe‐SA for both H_2_O_2_ and TMB substrates, respectively, indicating a significantly greater substrate affinity (Figure 2b; Figure S8 and Table S2, Supporting Information). The calculated *V*_max_ and catalytic efficiency (*k*_cat_/*K*_M_) of FeCu‐DA were 1.8‐ and 5.7‐fold greater, respectively, than those of Fe‐SA (Figure 2c; Table S2, Supporting Information), suggesting a faster catalytic velocity. Notably, FeCu‐DA exhibited a POD specific activity of 948.05 U mg^−1^, which surpassed that of both Fe‐SA and other reported nonnoble metal SAzymes (Figure 2d; Figure S9 and Table S3, Supporting Information). The enhanced POD activity of FeCu‐DA was further validated through electron paramagnetic resonance (ESR) analysis, which revealed more prominent characteristic peaks corresponding to 5,5‐dimethyl‐1‐pyrroline N‐oxide (DMPO) in the presence of H_2_O_2_ (Figure 2e), while these peaks were absent without H_2_O_2_, confirming POD activity. Interestingly, FeCu‐DA also demonstrated remarkable CAT and GSH‐OXD activities, leading to the oxidation of GSH in the presence of O_2_ and resulting in the production of H_2_O_2_ (Figure 2f,g). During a 9‐min interval, the intensity of 5,5′‐dithiobis‐(2‐nitrobenzoic acid) (DTNB) decreased to 34.5%, suggesting that FeCu‐DA consumed 65.5% of the GSH (Figure S10, Supporting Information). H_2_O_2_ production was verified through the utilization of an amplex red probe (Figure S11, Supporting Information). The primary antioxidant in tumor tissue, GSH, often hinders nanocatalytic therapies by neutralizing ROS. Therefore, FeCu‐DA overcomes this challenge by consistently generating a substantial quantity of cytotoxic ∙OH utilizing endogenous H_2_O_2_ while simultaneously inhibiting the action of endogenous GSH in neutralizing the produced ROS.

The photothermal effectiveness of single‐atom nanozymes (M‐N‐C), particularly Cu‐SAs, has been well established in previous studies, facilitating their application in photothermal therapy (PTT).^[^ ^17^ ^]^ FeCu‐DA exhibited a broad absorption band from 300 to 900 nm (Figure S12, Supporting Information). Upon near‐infrared (NIR) irradiation at 808 nm with a power density of 0.5 W cm^−2^, the temperature change of the FeCu‐DA solutions was more pronounced than that of the Fe‐SA solutions (Figure S13, Supporting Information). Specifically, a 20 µg mL^−1^ FeCu‐DA solution was heated to 52.9 °C after 9 min of NIR exposure, as confirmed by thermal imaging (Figure 2h). The temperature increase was also strongly correlated with both concentration and laser power (Figure S14, Supporting Information). The photothermal conversion efficiency (*η*) of FeCu‐DA was calculated to be 46.3% at 20 µg mL^−1^ (Figure 2i), outperforming most reported nanomaterials (Table S4, Supporting Information). FeCu‐DA demonstrated excellent thermal stability, maintaining consistent performance over five consecutive heating/cooling cycles under 808 nm laser irradiation (Figure 2j). These findings highlight the potential of FeCu‐DA as an effective agent for PTT because of its outstanding photothermal performance in the NIR range. Furthermore, the photothermal effect enhanced the production of ·OH, which was attributed to the increased POD and GSH‐OXD activities (Figure 2k; Figures S15 and S16, Supporting Information). This enhancement is driven by the elevated temperatures under 808 nm laser irradiation, which accelerates the reaction dynamics.^[^ ^18^ ^]^

As illustrated in Figure 2l, the obtained FeCu‐DA exhibited potent intratumoral H_2_O_2_ catalysis, generating highly cytotoxic ∙OH and O_2_ through its POD and CAT activities. Moreover, it facilitates the oxidation of GSH to produce H_2_O_2_ in the presence of O_2_ due to its GSH‐OXD activity. Taken together, the adjacent Fe and Cu sites on FeCu‐DA form an efficient cascade catalytic platform for sustained ∙OH generation and GSH depletion, thereby amplifying oxidative stress and exacerbating redox imbalance. Additionally, the exceptional photothermal effects of FeCu‐DA further enhance its cascaded catalytic activities.

### 2.3 Study of the POD Activity of FeCu‐DA via DFT Calculations

**Figure 3:**
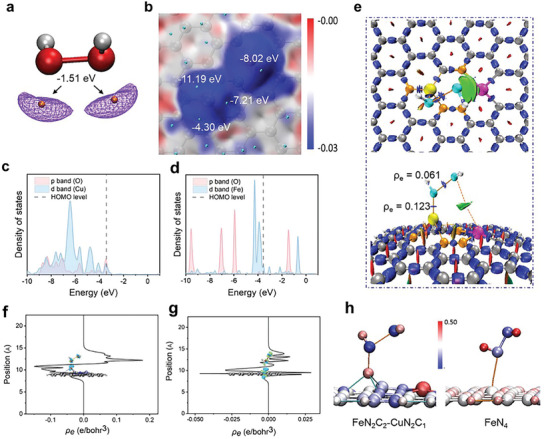
Density functional theory calculations. a) Electrostatic potential surface minima of H_2_O_2_ molecules. b) Local electron attachment energy (LEAE) of the FeCu‐DA surfaces. c,d) PDOS of H_2_O_2_/FeCu‐DA and H_2_O_2_/Fe‐SA with the FeN_4_ configuration. e) Interaction region indicator and electron density of selected bond critical points for H_2_O_2_/FeCu‐DA. f,g) Electron density difference for H_2_O_2_/FeCu‐DA and H_2_O_2_/Fe‐SA with the FeN_4_ configuration. h) Atomic dipole‐corrected Hirshfeld atomic charge of H_2_O_2_/FeCu‐DA and H_2_O_2_/Fe‐SA with the FeN_4_ configuration.

The high catalytic performance of FeCu‐DA was revealed through density functional theory (DFT) calculations. Since mimicking POD is a crucial step in this cascaded catalysis, we investigated the adsorption and activation of H_2_O_2_ on both the FeCu‐DA and Fe‐SA surfaces.^[^ ^19^ ^]^ The optimized model of FeCu‐DA was constructed on the basis of the coordination environment of its Fe and Cu centers as determined by XAS data (see supporting information for computational details). As revealed from the electrostatic potential surface minima in **Figure** **3**a, H_2_O_2_ is a strongly nucleophilic molecule that possesses lone‐pair electrons. Thus, the local electron attachment energy (LEAE) was evaluated as a local reactivity descriptor for predicting the interactions between nucleophilic molecules H_2_O_2_ and catalyst surfaces.^[^ ^20^ ^]^ The FeCu‐DA surface with a FeN_2_C_2_‐CuN_2_C_1_ configuration presented significantly lower surface minima near both the Fe and Cu sites, compared to the FeFe‐DA surface (FeN_2_C_2_‐FeN_2_C_1_ configuration) and Fe‐SA (FeN_2_C_2_ and FeN_4_ configurations) (Figure 3b; Figure S17, Supporting Information). This result suggests that both the Fe and Cu sites on the FeCu‐DA surface are highly reactive toward nucleophilic molecules such as H_2_O_2_. Furthermore, the partial density of states (PDOS) profile revealed a significant overlap between the *p* orbital of the O atom in H_2_O_2_ and the *d* orbital of the Cu atom on the FeCu‐DA surface (Figure 3c), indicating potential chemical bonding interactions between H_2_O_2_ and the Fe/Cu active sites. In contrast, no such overlap was observed between H_2_O_2_ on FeFe‐DA or Fe‐SA, indicating weaker interactions (Figure 3d; Figure S18, Supporting Information). The interaction region indicator (IRI) calculation suggested that H_2_O_2_ can be adsorbed onto the surface of FeCu‐DA through both chemical bonding interactions and van der Waals interactions (Figure 3e),^[^ ^21^ ^]^ which significantly enhances the activation of O─O bonds in H_2_O_2_ on the surface of FeCu‐DA. In contrast, on the Fe‐SA surfaces, the adsorption of H_2_O_2_ was exclusively driven by van der Waals interactions without significant activation of the O─O bond (Figure S19, Supporting Information). The electron transferred from the Fe/Cu active sites to H_2_O_2_ was more pronounced than the transfer from the FeN_4_ active sites (Figure 3f,g). The atomic dipole‐corrected Hirshfeld atomic charge (ADCH) further revealed a net charge of −0.344 for H_2_O_2_ on the surface of FeCu‐DA (Figure 3h),^[^ ^22^ ^]^ which significantly exceeded that of H_2_O_2_ on the Fe‐SA surface with the FeN_4_ configuration (−0.005). This suggests that the electron transferred between the catalyst surface and H_2_O_2_ molecules likely promotes the activation of O─O bonds. Atoms‐in‐Molecules (AIM) analysis^[^ ^23^ ^]^ also revealed that the electron density at the bond critical point (BCP) of the O─O bond on the FeCu‐DA surface (0.061) was significantly lower than that on the FeFe‐DA and Fe‐SA surfaces (Figure 3e; Figure S19, Supporting Information). In summary, compared with the FeFe‐DA and Fe‐SA surfaces, the FeCu‐DA surface results in superior H_2_O_2_ activation, effectively facilitating the subsequent decomposition of H_2_O_2_ molecules.

### 2.4 Therapeutic Effect of the FeCu‐DA Nanozyme In Vitro

Arginine‐glycine‐aspartic acid (RGD) plays a crucial role in specifically binding with integrins that are overexpressed on both the tumor neovasculature and tumor cells.^[^ ^24^ ^]^ The incorporation of RGD onto material surfaces enhances their biocompatibility and targeting capabilities. After surface modification with RGD, the FeCu‐DA particles exhibited uniform sizes, and their surface charge shifted from 26.17 to −22.82 mV, facilitating improved internalization by cancer cells (Figure S20, Supporting Information). Importantly, their catalytic performance and photothermal effects remained unaffected (Figure S21, Supporting Information).

**Figure 4:**
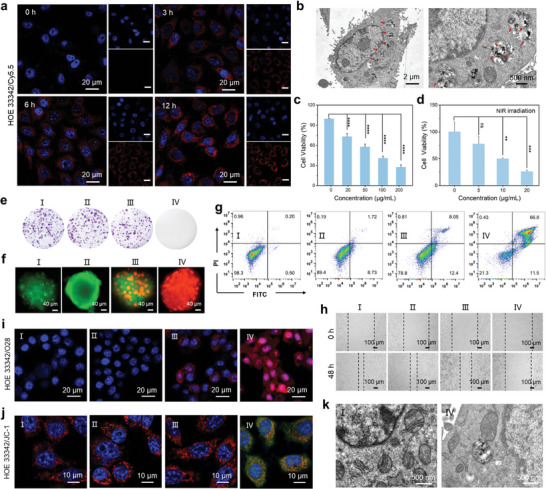
Cytotoxicity properties of FeCu‐DA. a) Cellular uptake of Cy5.5‐labeled FeCu‐DA by 4T1 cells after various incubation durations. b) Bio‐TEM image of 4T1 after incubation with FeCu‐DA. c) MTT assay of 4T1 cells treated with different concentrations of FeCu‐DA. d) MTT assay of 4T1 cells treated with FeCu‐DA and NIR irradiation (808 nm, 0.5 W cm^−2^). e) Colony formation ability of 4T1 cells subjected to different treatments. f) Tumor sphere formation assay of 4T1 cells. Live and dead cells are stained with green and red fluorescence, respectively. g) Flow cytometry apoptosis assay of 4T1 cells subjected to different treatments. h) Wound assay of 4T1 cells in various groups. i) Detection of intracellular ·OH levels in various groups. j) JC‐1 analysis of cells as a measure of mitochondrial depolarization. The color shows JC‐1 aggregates (red) in the mitochondria and JC‐1 monomers (green) in the cytosol. k) Bio‐TEM image of 4T1 cells after treatment with FeCu‐DA+NIR. Treatment groups I: Control, II: NIR, III: FeCu‐DA, and IV: FeCu‐DA+NIR. All quantitative data are presented as the means ± SDs (*n* = 4), and *p* values were calculated via one‐way ANOVA: ^*^ *p* < 0.05, ^**^ *p* < 0.01, ^***^ *p* < 0.001, ^****^ *p* < 0.0001, and no significant difference (ns).

To confirm the internalization of FeCu‐DA by 4T1 cells, we labeled FeCu‐DA with cyanine 5.5 (Cy5.5) dye and incubated it with 4T1 cells (**Figure** **4**a). The intensity of red fluorescence from Cy5.5 in the cytoplasm increased over time, indicating successful internalization of FeCu‐DA‐Cy5.5 by 4T1 cells. Flow cytometric analysis was also utilized to quantify endocytosis efficiency on the basis of fluorescence intensity (Figure S22, Supporting Information). The results revealed that the fluorescence intensity in 4T1 cells treated with Cy5.5‐labeled FeCu‐DA for 12 h was 9.2 times greater than that in untreated cells. Additionally, TEM analysis demonstrated that the FeCu‐DA particles were primarily localized in endosomes or lysosomes (Figure 4b), suggesting that these nanoparticles were internalized via phagocytosis/micropinocytosis.^[^ ^25^ ^]^ As shown in Figure 4c, the viability of the 4T1 cells decreased with increasing concentrations of FeCu‐DA, with an IC_50_ of 67.6 µg mL^−1^. Notably, at a concentration of 200 µg mL^−1^, the viability of 4T1 cells decreased to 27.6%. Treatment with FeCu‐DA at a low dosage of 20 µg mL^−1^ resulted in a reduction in tumor cell viability to 73.3%. Furthermore, upon exposure to NIR irradiation, the viability of 4T1 cells significantly decreased to 26.6%. (Figure 4d). The colony formation assay further confirmed the consistent inhibitory effect on 4T1 cells under the combined treatment of FeCu‐DA and NIR irradiation (Figure 4e). To avoid administering large doses of materials, we selected 20 µg mL^−1^ as the minimum effective concentration for further studies.

The calcein‐AM/PI costaining fluorescence images in Figure 4f displayed intense red fluorescence when FeCu‐DA and NIR irradiation were introduced, indicating high lethality toward 3D multicellular tumor spheroids (MTSs). Flow cytometry analysis supported these findings by confirming a high apoptosis rate in treated 4T1 cells (Figure 4g). A scratch assay was conducted to investigate the migration ability of treated 4T1 cells. As shown in Figure 4h, the wound healing process was inhibited when either FeCu‐DA alone or in combination with NIR irradiation was applied, whereas the control and NIR groups exhibited rapid wound healing. This observation strongly suggests a significant suppression of the proliferation and migratory capacities of 4T1 cells.

As shown in Figure 4i, treatment with FeCu‐DA and NIR irradiation resulted in a remarkable increase in the intracellular ∙OH level, as evidenced by the pronounced red fluorescence intensity in the 4T1 cells. Elevated ROS levels are known to induce tumor cell apoptosis through mitochondrial dysfunction.^[^ ^26^ ^]^ To investigate this further, we used JC‐1 staining. JC‐1 emits red fluorescence when it aggregates on intact mitochondrial membranes and emits green fluorescence from monomers on disrupted mitochondrial membranes. We observed a decrease in red fluorescence and an increase in green fluorescence (Figure 4j), indicating the perturbation of mitochondrial membranes and depolarization of the membrane potential under the combined treatment of FeCu‐DA and NIR irradiation. Additionally, the TEM image in Figure 4k revealed both volume shrinkage and the disappearance of cristae within the mitochondria. Furthermore, a decrease in the expression of heat shock protein 70 (HSP70) was noted in 4T1 cells treated with FeCu‐DA and NIR irradiation (Figure S23, Supporting Information), suggesting the maintenance of mild PTT efficacy.

**Figure 5:**
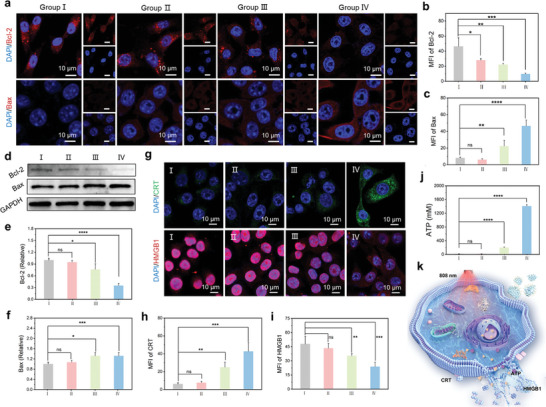
The mechanism of the apoptosis‐ and PTT‐induced release of DAMPs from 4T1 tumor cells in vitro. a) Confocal images of Bcl‐2 and Bax in 4T1 cells after various treatments. b,c) Analysis of the fluorescence intensity shown in Figure 5a. d) Western blot analysis of the protein expression of Bax and Bcl‐2 in 4T1 cells after various treatments. e,f) Analysis of the gray values in Figure 5d. g) Confocal images of CRT and HMGB1 in 4T1 cells after various treatments and analysis of the fluorescence intensity. h, i) Analysis of the fluorescence intensity shown in Figure 5g. j) ATP release from 4T1 cells after various treatments. k) Scheme for the induction of ICD. Treatment group I: Control; group II: NIR; group III: FeCu‐DA; and group IV: FeCu‐DA+NIR. All the quantitative data are presented as the means ± SDs (*n* = 3). *p* values were calculated via one‐way ANOVA: ^*^ *p* < 0.05, ^**^ *p* < 0.01, ^***^ *p* < 0.001, ^****^ *p* < 0.0001, and no significant difference (ns).

B‐cell lymphoma‐2 (Bcl‐2) family proteins play crucial roles in regulating apoptosis via the mitochondrial pathway.^[^ ^27^ ^]^ Dysregulation of antiapoptotic proteins, such as Bcl‐2, in various malignant cancer cells disrupts the expression of proapoptotic proteins such as Bax.^[^ ^28^ ^]^ These proteins control mitochondrial outer membrane permeability (MOMP), thereby activating caspase‐3 during apoptosis. To validate this signaling pathway, we assessed the expression of Bcl‐2, Bax and caspase‐3 via both confocal laser scanning microscopy and western blotting. The immunofluorescence images in **Figure** **5**a,b revealed a significant decrease in the Bcl‐2 fluorescence intensity in 4T1 cells treated with FeCu‐DA and NIR irradiation. Concurrently, we observed a notable increase in Bax intensity (Figure 5a,c). These findings were further supported by the western blot results, which demonstrated a consistent trend in the expression of Bcl‐2 and Bax (Figure 5d–f). The activation of caspase‐3 was also confirmed via western blotting (Figure S24, Supporting Information). Overall, these results indicate that the combined treatment effectively induces apoptosis in 4T1 cells by downregulating the expression of antiapoptotic proteins, promoting the release of proapoptotic proteins, and subsequently activating caspase family proteins.

In addition to apoptosis, ICD can be induced through photothermal ablation and increased ROS levels. This process leads to the release of DAMPs, which activate systemic anticancer immune responses.^[^ ^1b^ ^]^ To explore the potential of FeCu‐DA treatment in triggering ICD in tumor cells, the secretion of diverse DAMPs was examined through cellular experiments. Treatment with FeCu‐DA and NIR irradiation resulted in a notable relocation of calreticulin (CRT), as indicated by the robust green fluorescence signal originating from the plasma membrane (Figure 5g,h). During ICD, CRT rapidly translocates to the cytomembrane and integrates into the phospholipid bilayer, generating an “eat me” signal that recruits macrophages and dendritic cells (DCs).^[^ ^4^ ^]^ Additionally, high mobility group box protein 1 (HMGB1) was secreted from the nucleus region into the cytoplasm under this combined treatment (Figure 5g,i), suggesting rupture and disintegration of the nuclear envelope. Adenosine triphosphate (ATP), another important DAMP, can leak and propagate during the preapoptotic stage through the activation of the NLRP3 inflammasome. As shown in Figure 5j, the release of ATP was 7.0‐fold greater in the 4T1 cells treated with both FeCu‐DA and NIR irradiation than in the FeCu‐DA group. These observations confirm that FeCu‐DA can effectively induce ICD, initiating a distant and enduring immune response against tumors (Figure 5k). Notably, the loading dose of FeCu‐DA (20 µg mL^−1^) is notably lower than that of reported SAzymes (≈200 µg mL^−1^).

### 2.5 Antitumor Efficacy of the FeCu‐DA Nanozyme In Vivo

**Figure 6:**
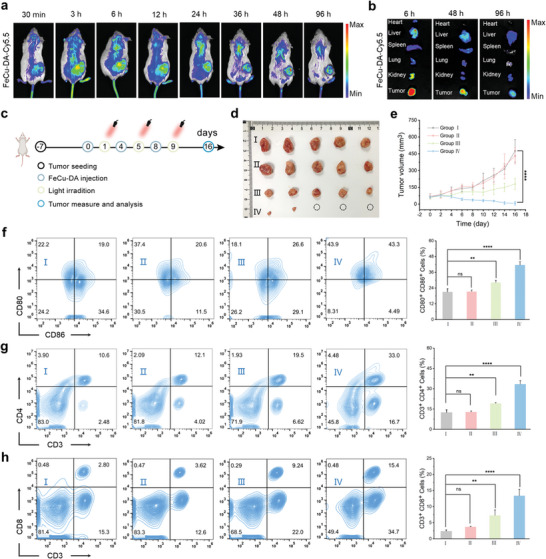
Antitumor effects of synergistic FeCu‐DA PTT/CDT therapies in vivo. a, b) Time‐dependent biodistribution of Cy5.5‐labeled FeCu‐DA in tumor‐bearing mice and biodistribution in major organs and tumors at specific time points postinjection. c) Schematic illustration of the treatment schedule for BALB/c mice bearing 4T1 tumors. d) Photographs of excised tumors on day 16. e) Tumor growth profile during the therapeutic period of 16 days. f) Representative flow cytometric plots of CD86^+^ and CD80^+^ T‐cell populations in the spleen after various treatments. g) Representative flow cytometric plots of CD3^+^ CD4^+^ T‐cell populations in the spleen after various treatments. h) Representative flow cytometric plots of CD3^+^ CD8^+^ T‐cell populations in the spleen after various treatments. Treatment group I: Control; group II: NIR; group III: FeCu‐DA; and group IV: FeCu‐DA+NIR. All the quantitative data are presented as the means ± SDs (*n* = 5). *p* values were calculated via one‐way or two‐way ANOVA: ^*^ *p* < 0.05, ^**^ *p* < 0.01, ^***^ *p* < 0.001, ^****^ *p* < 0.0001, and no significant difference (ns).

Building upon the notable immune activation and antitumor effects observed in vitro, we further evaluated the anticancer efficacy of FeCu‐DA‐mediated photothermal therapy/chemodynamic therapy (PTT/CDT) and its role in establishing an immune response in vivo. The tissue distribution of FeCu‐DA was tracked via the dye Cy5.5 for fluorescence imaging. As shown in **Figure** **6**a, the fluorescence intensity in the tumor region began to increase at 3 h postinjection and remained detectable for up to 96 h, indicating that FeCu‐DA accumulated in the tumor tissue. To further investigate the biodistribution and metabolism of FeCu‐DA, we measured fluorescence in major organs and tumor tissues excised at specific time points postinjection. As shown in Figure 6b, the fluorescence intensity was notably high in the tumor, liver, and kidney, suggesting that FeCu‐DA effectively accumulated in tumor tissue and was primarily cleared through the hepatic and renal pathways. This excretion pattern is consistent with observations in healthy mice (Figure S25, Supporting Information) and corroborates findings from related studies.^[^ ^29^ ^]^

The tumor‐bearing mice were randomly divided into four groups (five per group) and subjected to various treatments (I: Control; II: NIR; III: FeCu‐DA; IV: FeCu‐DA+NIR). As shown in Figure 6c, groups III and IV received intravenous FeCu‐DA (10 mg kg^−1^) every three days for a total of three doses. Furthermore, after each intravenous administration for 12 and 24 h, groups II and IV were exposed to NIR irradiation at a laser density of 0.5 W cm^−2^ for 9 min. As illustrated in Figure 6d,e, the control and NIR groups exhibited continued significant tumor growth. In contrast, the group III presented a tumor growth suppression rate of 54.4% on the basis of the tumor volume, highlighting the effectiveness of FeCu‐DA. Notably, the tumor growth suppression rate further increased to 98.4% in group IV, emphasizing the synergistic effect of combining FeCu‐DA with NIR irradiation. Histological analysis (Figure S26, Supporting Information) revealed significantly increased levels of nuclear pyknosis, intense staining, and necrosis in group IV, whereas Ki67 (Figure S27, Supporting Information) confirmed a marked reduction in tumor cell proliferation. Moreover, the combination of FeCu‐DA and NIR irradiation led to the highest levels of CRT expression (Figure S28, Supporting Information), strongly indicating the induction of ICD.

To gain insight into the underlying mechanism driving the systemic immune response against tumors triggered by FeCu‐DA‐mediated PTT/CDT, we investigated the maturation process of DCs in the spleen, the largest immune organ in the body. As shown in Figure 6f, the proportion of mature DCs (CD80^+^ CD86^+^) in the spleen significantly increased in treatment groups III and IV. Specifically, group III exhibited a 1.4‐fold increase, and group IV presented a 2.3‐fold increase compared with group I. DCs play a pivotal role as antigen‐presenting cells (APCs), orchestrating the proliferation and differentiation of helper T cells (CD3^+^CD4^+^ T cells) and tumor‐specific cytotoxic T cells (CD3^+^CD8^+^ T cells), thereby effectively eliminating malignant cells.^[^ ^30^ ^]^ As a result, groups III and IV presented 1.8‐ and 3.1‐fold greater proportions of CD3^+^ CD4^+^ T cells in the spleen than group I did (Figure 6g). Furthermore, groups III and IV exhibited remarkable 3.3‐ and 5.5‐fold increases in the population of CD3^+^ CD8^+^ T cells compared with group I (Figure 6h). In addition to the T‐cell response, the polarization of tumor‐associated macrophages (TAMs) was also evaluated (Figure S29, Supporting Information). The results indicated a significant decrease in the proportion of M2‐type macrophages (CD206^+^ CD86^−^) from 20.7% in group I to 2.1% and 1.1% after treatment in groups III and IV, respectively. Simultaneously, there was a notable increase in the population of M1 macrophages (CD86^+^ CD206^−^) from 32.9% in group I to 41.4% in group III and 51.0% in group IV. These findings suggest a substantial increase in the proinflammatory macrophage response. These findings suggest that the treatment effectively shifted TAM polarization toward the proinflammatory M1 phenotype, further enhancing the antitumor response.

The biosafety of FeCu‐DA was thoroughly assessed. Figure S30 (Supporting Information) shows a slight increase in the body weight of all treated mice, indicating that intravenous administration of FeCu‐DA has low toxicity and excellent biocompatibility. Additionally, no significant hemolysis was observed at a high concentration of 200 µg mL^−1^ (Figure S31, Supporting Information). Comprehensive blood analyses were conducted to assess key biomarkers, and the results revealed minimal signs of inflammation, liver toxicity, or kidney toxicity (Figure S32, Supporting Information). Finally, major organs extracted from the treated mice were subjected to H&E staining (Figure S33, Supporting Information), which revealed no observable tissue damage. These findings underscore the favorable safety profile of FeCu‐DA, highlighting its potential for future clinical applications.

### 2.6 Combination of FeCu‐DA‐Induced ICD and αPD‐L1 for Enhanced Immunotherapy

**Figure 7:**
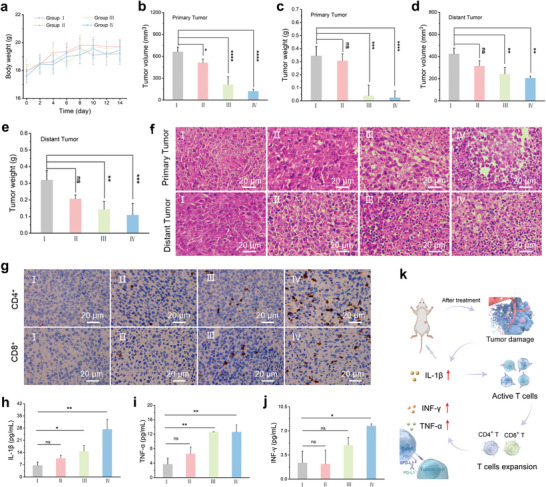
Antitumor effects of synergistic PTT/CDT therapies with FeCu‐DA and αPD‐L1 against distant tumors. a) Changes in body weight during treatment (*n* = 4). b,c) Changes in the tumor volume and weight of the primary tumor after various treatments (*n* = 4). d,e) Changes in the tumor volume and weight of distant tumors after various treatments (*n* = 4). f) H&E staining of primary and distant tumor tissues. g) Immunohistochemistry (IHC) images showing CD4^+^ T cells and CD8^+^ T cells. h–j) The levels of IL‐1*β*, TNF‐*α*, and IFN‐*γ* in the serum of different groups were measured via ELISA (*n* = 3). k) Schematic illustration of the antitumor immune effect in vivo. Treatment groups I: Control, II: αPD‐L1, III: FeCu‐DA+NIR, IV: FeCu‐DA+NIR+ αPD‐L1. The data are shown as the means ± SDs, and *p* values were calculated via one‐way ANOVA: ^*^ *p* < 0.05, ^**^ *p* < 0.01, ^***^ *p* < 0.001, ^****^ *p* < 0.0001, and no significant difference (ns).

Distal metastasis remains the primary cause of failure in clinical tumor treatment. After confirming the synergistic induction of ICD through FeCu‐DA‐mediated PTT/CDT, we sought to determine whether the combination of ICD and αPD‐L1 could enhance the immune response against distant tumors. Importantly, there was no negligible fluctuation in body weight among the treatment groups during the study period (**Figure** **7**a), indicating the safety of these interventions. As anticipated, treatment with FeCu‐DA and NIR irradiation effectively suppressed tumor growth at both the primary and distant sites (Figure 7b–e). The addition of an αPD‐L1 further increased the tumor suppression rates at both sites (Figure 7b–e). Histological analysis of excised tumor tissues from both sites revealed significantly increased nuclear pyknosis, intense staining, and necrosis (Figure 7f), indicating effective tumor cell death.

To further evaluate the immune response, distant tumor tissue was subjected to immunofluorescence staining for CD8^+^ and CD4^+^ T cells. As shown in Figure 7g and Figure S34 (Supporting Information), the FeCu‐DA+NIR+ αPD‐L1 treatment group presented the highest level of CD8^+^ and CD4^+^ T‐cell expression, confirming the activation of the immune response. The increased presence of these T cells contributes to tumor immunity by producing and releasing perforin, cytokines and other effector molecules.^[^ ^2^, ^30^ ^]^ Additionally, the serum expression levels of interleukin (IL)‐1*β*, tumor necrosis factor (TNF)‐*α*, and interferon (IFN)‐*γ* were significantly elevated in the FeCu‐DA+NIR+ αPD‐L1 treatment group (Figure 7h–j). These cytokines, which are released by immune cells, further indicate a robust antitumor immune response. Collectively, these findings confirm that the combination of synergistic ICD and immune checkpoint blockade (ICB) therapy significantly enhances the antitumor immune effect (Figure 7k).

## 3 Conclusion

In summary, we developed a cascaded FeCu‐DA with Fe and Cu sites for ICD‐induced cancer immunotherapy. The coordination states of Fe and Cu single atoms were determined through XANES and EXAFS investigations. FeCu‐DA exhibited excellent photothermal effects and effectively mimicked the functions of POD, CAT, and GSH‐OXD, enabling enhanced cascaded catalysis under NIR irradiation. Theoretical calculations revealed that the high POD property originated from strong interactions between the surface of FeCu‐DA and H_2_O_2_ due to significant orbital overlap. In vitro and in vivo experiments confirmed the efficacy of FeCu‐DA in inducing potent ICD and its remarkable therapeutic effects on breast cancer under NIR irradiation. Furthermore, the combination of FeCu‐DA and αPD‐L1 demonstrated synergistic enhancement of treatment outcomes when paired with NIR irradiation. The present study not only offers valuable insights for the design of highly efficient dual‐atom nanozymes but also demonstrates their potential in enhancing cancer immunotherapy through robust induction of ICD and ICB therapies.

## 4 Experimental Section

### Preparation of FeCu‐DA

A transparent solution was prepared by dissolving 4.0 g of citric acid, 4.0 g of NH_4_Cl, 0.26 g of Fe(NO_3_)3⋅9H_2_O, and 0.12 g of Cu(NO_3_)_2_ in 12 mL of deionized water. Next, 1.2 g of SiO_2_, which served as a template, was uniformly dispersed into the solution via ultrasonication and vigorously stirred at room temperature for 12 h. The homogeneous solution was then freeze‐dried to yield a solid powder, which was ground for 15 min and then carbonized at 900 °C for 3 h under an Ar atmosphere with a heating rate of 10 °C min^−1^. The resulting black carbonized product was dispersed in a 5 wt.% aqueous HF solution at room temperature for 10 h to completely etch the SiO_2_ template. Finally, FeCu‐DA was obtained by washing and drying the material at 80 °C.

### Photothermal Properties

The photothermal performance of FeCu‐DA was assessed by irradiating 2.0 mL aqueous solutions with varying FeCu‐DA concentrations (0, 5, 10, and 20 µg mL^−1^) with an 808 nm laser at power densities of 0.38, 0.50, and 0.55 W cm^−2^, respectively, for 9 min. The temperature changes were recorded via an infrared (IR) thermal camera. The photothermal stability was examined over five cycles of alternating laser on/off irradiation. The photothermal conversion efficiency was calculated according to the procedure detailed in Supporting Information.

### Enzymatic Activities

POD activity was evaluated via a typical TMB chromogenic reaction. Briefly, 100 µL of TMB solution (10 mm), 300 µL of H_2_O_2_ (10 mm) and varying volumes of FeCu‐DA solution (100 µg mL^−1^) were added to an acetic acid/sodium acetate buffer solution (pH 4.0) to a final volume of 1 mL. The mixture was incubated in the dark at 37 °C for 10 min, and the absorbance of the oxidized TMB was measured via UV‒vis absorption spectroscopy. The procedure was repeated to compare the absorbance values at 37 and 50 °C. The GSH‐OXD activity was determined via a colorimetric method with DTNB as a chromogenic substrate. A typical assay was conducted by adding 20 µL of FeCu‐DA (1 mg mL^−1^) and 50 µL of GSH solution (20 mM) to a PBS buffer solution (pH 6.0) to a final volume of 1 mL. The mixture was incubated at 37 °C for 0, 1, 3, 6, or 9 min. Subsequently, 100 µL of the reaction mixture was diluted with a PBS buffer solution (pH 8.0) to 1 mL, followed by the addition of 20 µL of DTNB solution (5 mm). The absorbance of the solution was recorded via a UV‒vis spectrophotometer for analysis. This process was repeated to compare the absorbance values at 37 and 50 °C. The CAT activity was determined by measuring the dissolved oxygen content in a PBS solution (pH 6.0) via a portable analyzer. 200 µL of FeCu‐DA (1 mg mL^−1^) and 10 µL of H_2_O_2_ (10 m) were added to a PBS buffer solution (pH 6.0) to a final volume of 10 mL. The interval was 20 s for detection for 15 min.

### Surface Modification of FeCu‐DA Nanozymes

First, 10 mg of FeCu‐DA was added to 10 mL of iRGD‐PEG‐DSPE solution (1 mg mL^−1^) and incubated overnight. After washing, the RGD‐modified FeCu‐DA was redispersed in a DMSO solution and mixed with 50 µL of trisulfo‐CY5.5 NHS solution (1 mg mL^−1^) overnight for labeling. The sample was then washed twice to remove any unbound trisulfo‐CY5.5 NHS. Finally, the Cy5.5‐labeled FeCu‐DA was redispersed in PBS for subsequent experimental use.

### Cellular Uptake of FeCu‐DA Nanozymes

4T1 cells were seeded in plates and incubated for 24 h. Then, 20 µL of Cy5.5‐labeled FeCu‐DA (1  mg mL^−1^) was added to the cells and incubated for various durations. Cellular fluorescence was recorded via confocal laser scanning microscopy (CLSM) to visualize the internalization and distribution of FeCu‐DA. Additionally, flow cytometry (FCM) was performed to quantify the fluorescence intensity and evaluate the efficiency of FeCu‐DA uptake at different time points.

### Statement of Ethical Approval for the Animal Experiments

Female BALB/c mice were purchased from Spfbiotech Co., Ltd. (Beijing, China) and housed at the Experimental Animal Center at Guangxi Medical University. All experimental procedures involving animals were conducted in accordance with protocols approved by the Ethical Committee of Guangxi Medical University Cancer Hospital (approved number: KY‐2022‐371).

### In Vivo Distribution of FeCu‐DA

4T1 cells were subcutaneously inoculated into BALB/c mice. Once the tumors reached ≈200 mm^3^ in size, Cy5.5‐labeled FeCu‐DA was intravenously administered at a dose of 10 mg kg^−1^. The mice were then anesthetized and imaged at multiple time points via a small animal live imager to monitor the real‐time biodistribution of FeCu‐DA. At 6, 48, and 96 h postadministration, the mice were sacrificed, and their heart, liver, spleen, lungs, kidneys, and tumor tissues were dissected and collected. Ex vivo fluorescence imaging was performed on these tissues to capture fluorescence intensity, enabling the assessment of FeCu accumulation and distribution across various organs and tumors.

### Antitumor Therapy In Vivo

The 4T1 tumor‐bearing mouse model was established by subcutaneously injecting 8 × 10^6^ 4T1 cells suspended in 100 µL of PBS into female BALB/c mice. The mice were randomly divided into four treatment groups: Control (I), NIR (II), FeCu‐DA (III), and FeCu‐DA + NIR (IV). On day 0, the mice in groups III and IV received an intravenous injection of an FeCu‐DA solution on days 0, 4, and 8 at a dose of 10 mg kg^−1^, whereas the mice in group I received a PBS solution. Twelve hours postinjection, the mice in groups II and IV underwent laser irradiation at a wavelength of 808 nm and an intensity of 0.5 W cm^−2^ for 9 min. Mouse body weight and tumor diameter were measured every other day over a 16‐day period. On day 16, the tumors and major organs were excised from the mice in each group for histological evaluation via hematoxylin‒eosin (H&E) staining.

### Evaluation of the Effects of ICD and Immune Checkpoint Blockade Therapy on Distant Tumors

Female BALB/c mice were subcutaneously injected with a cell suspension (8 × 10^6^ on the left flank and 5 × 10^6^ on the right flank) to establish primary and distant tumors, respectively. The 4T1 tumor‐bearing mice were randomly assigned to four groups: the Control (I), αPD‐L1 (II), FeCu‐DA + NIR (III), and FeCu‐DA + NIR + αPD‐L1 (IV) groups. The mice in groups III and IV received intravenous injections of FeCu‐DA (5 mg kg^−1^) on days 1, 3, and 5. Twelve hours postinjection, laser irradiation (808 nm, 0.5 W cm^−2^, 9 min) was applied to the primary tumors in groups III and IV. Groups II and IV were intraperitoneally administered αPD‐L1 (200 µg mL^−1^) on days 2, 4, and 6. Mouse body weights and tumor sizes were recorded every other day over a 14‐day observation period. On day 14, the mice were euthanized. The left‐sided (primary) tumors were collected for HE staining as well as immunohistochemistry analysis. Serum was extracted for enzyme‐linked immunosorbent assay (ELISA) analysis to determine the levels of IL‐1β, TNF‐α, and IFN‐γ. All major organ and blood samples were collected to assess potential toxicity. In addition, fresh spleen tissues were obtained and homogenized through a 200‐mesh nylon screen, and the resulting cell suspension was processed via a mouse spleen mononuclear cell isolation kit. After centrifugation at 800 g for 30 min, mononuclear cells were stained with anti‐CD3 (PC5.5), anti‐CD4 (Cy7), anti‐CD4 (Cy7) anti‐CD8 (AF647), anti‐CD11b (AF‐488), anti‐CD206 (AF647), anti‐CD80 (PE), and anti‐CD86 (Cy7) antibodies according to the BD Pharmingen manufacturer's protocol. Finally, the stained cells were analyzed via a flow cytometer, and the data were processed with FlowJo software to evaluate immune responses.

### Statistical Analysis

To ensure the accuracy of the experiments, at least three replicates were performed. All test data are presented as the means ± standard deviations, and the statistical analyses were performed via Origin 2018 and GraphPad Prism 9.0. One‐way or two‐way ANOVA was used for data with multiple comparison groups. In this study, statistical significance was defined as ^*^ *p* < 0.05, ^**^ *p* < 0.01, ^***^ *p* < 0.001, ^****^ *p* < 0.001, and no significant difference (ns).

## Conflict of Interest

The authors declare no conflict of interest.

## Supporting information



Supporting Information

## Data Availability

The data that support the findings of this study are available from the corresponding author upon reasonable request.
