# Supplementary material for: A Synergistic Dual‐Atom Sites Nanozyme Augments Immunogenic Cell Death for Efficient Immunotherapy
Source: Adv Sci (Weinh). 2024 Dec 24;12(7):2414734. doi: 10.1002/advs.202414734 (PMC11831451; doi:10.1002/advs.202414734)
Supplement: Supplementary file 1 — Supporting Information [file ADVS-12-2414734-s001.docx]

Supporting Information

**A Synergistic Dual-Atom Sites Nanozyme Augments Immunogenic Cell Death for Efficient Immunotherapy**

Shipeng Ning*, Zeyuan Zhang, Yujing Ren, Yaxin Hou, Dan Li, Jingqi Chen, Yujie Zhai, Kelong Fan*, Weiqing Zhang*

S. Ning

Department of Breast Surgery, The Second Affiliated Hospital of Guangxi Medical University, Nanning, 530000, China

E-mail: [nspdoctor@sr.gxmu.edu.cn](mailto:nspdoctor@sr.gxmu.edu.cn)

Z. Zhang, D. Li, J. Chen, Y. Zhai, W. Zhang

Department of Research, Guangxi Medical University Cancer Hospital, Guangxi Medical University, Nanning 530021, China

University Engineering Research Center of Oncolytic & Nanosystem Development, 530021, Guangxi

E-mail: [zhangweiqing@tjut.edu.cn](mailto:zhangweiqing@tjut.edu.cn)

Z. Zhang

West China School of Medicine, Sichuan University, Chengdu 610041, China

Y. Ren

Interdisciplinary Research Center of Biology & Catalysis, School of Life Sciences, Northwestern Polytechnical University Xi’an 710072, China

Y. Hou, K. Fan

CAS Engineering Laboratory for Nanozyme, Key Laboratory of Biomacromolecules (CAS), CAS Center for Excellence in Biomacromolecules, Institute of Biophysics, Chinese Academy of Sciences, Beijing 100101, China.

E-mail: [fankelong@ibp.ac.cn](mailto:fankelong@ibp.ac.cn)

K. Fan

Nanozyme Laboratory in Zhongyuan, Henan Academy of Innovations in Medical Science, Zhengzhou, Henan, 451163, China.

**Experimental Section/Methods**

1. Materials

Ar was purchased from Nanning Air Gas Co., Ltd. (China). Copper dinitrate (Cu(NO_3_)_2_) was purchased from Shanghai, Macklin Biochemical Technology Co., Ltd. (China). Citric acid monohydrate (C₆H₈O₇) and hydrofluoric acid (HF) were purchased from Sinopharm Group Chemical Reagent Co., Ltd. (China). Silicon dioxide (SiO_2_), iron nitrate nonahydrate (Fe(NO_3_)_3_•9H_2_O), ammonium chloride (NH_4_Cl), dimethyl sulfoxide (DMSO), 3,3ʹ,5,5ʹ-tetramethylbenzidine (TMB) and 5,5'-dithiobis-(2-nitrobenzoic acid) (DTNB) were purchased from Shanghai Aladdin Co., Ltd. (China). H_2_O_2_ (30%) was purchased from Tianjin Obo Kai Chemical Co., Ltd. NaAc buffer was purchased from Shanghai Yuanye Biotechnology Co., Ltd. (China). PBS buffer and a hydroxyl free radical scavenging capacity assay kit were purchased from Beijing Solarbio Co., Ltd. (China). iRGD-PEG-DSPE and trisulfo-CY5.5 NHS were purchased from Tansh-Tech (China). A calcein AM/PI cell double-staining kit, Hoechst 33342, 5,5′,6,6′-tetrachloro-1,1′,3,3′-tetraethylbenzimidazolyl-carbocyanine chloride (JC-1), 3-(4,5)-dimethylthiahiazo (-z-y1)-3,5-phenytetrazoliumromide (MTT) and an ATP assay kit were purchased from Beyotime (China). The Annexin V-FITC/PI apoptosis assay mixture and O28 were purchased from Bestbio Co., Ltd. (China). Anti-Tubulin, anti-GAPDH, anti-Bcl-2, anti-Bax, anti-HSP70, anti-CRT and anti-HMGB1 antibodies were purchased from ABclonal Co., Ltd. (China). Anti-calreticulin was purchased from Abcam Co., Ltd. (UK). Anti-CD3 (PC5.5), anti-CD4 (Cy7), anti-CD8 (AF647), anti-CD11b (AF-488), anti-CD206 (AF647), anti-CD80 (PE), and anti-CD86 (Cy7) antibodies were purchased from Becton, Dickinson and Company (USA). Fetal bovine serum (FBS) and Dulbecco’s modified Eagle’s medium (DMEM) were purchased from Gibco (USA). Streptomycin, penicillin, and trypsin were obtained from Solarbio (China). The aqueous solution used in the experiment was prepared from the deionized water of a Milli-Q water purification system.

2. Methods

*2.1 Computational methods*

All the DFT calculations conducted in this work were performed via the CP2K package,^[S1]^ which is based on the PBE functional and a hybrid Gaussian/Plane-Wave scheme (GPW). ^[S2]^ Core electrons are described by GTH pseudopotentials. ^[S3]^ The wave functions were expanded in optimized double-ζ Gaussian basis sets^[S4],^ and the plane waves were expanded with a cutoff energy of 400 Rydberg. The theoretical models for the FeCu-DA and Fe-SA surfaces were constructed with Fe and Cu atoms supported on a graphite-like structure with some C atoms replaced by N atoms (based on the coordination environment of the Fe and Cu centers, as determined from XAS data). The dangling bonds at the edge of the catalyst support were saturated with H atoms, which were preoptimized and fixed at their position during the subsequent calculations. The construction of the input files and postprocessing of the CP2K calculated data were carried out via the Multiwfn program. ^[S5]^

*2.2 The calculation of specific activity*

The POD specific activity of FeCu-DA was determined according to the modified method specified in the national standard of China (GB/T 37966-2019). Typically, TMB and H_2_O_2_ solutions were added into NaAc-HAc buffer solution (pH 4.0) containing various concentrations of FeCu-DA. Then, the absorbance of the reaction solution at 652 nm (A_652_) was immediately recorded every 20 s for 10 min. The A_652_ against the reaction time was plotted and the POD active unit number (b_FeCu-DA_) was calculated according to equation:

b_FeCu-DA_ = 𝑉/ (𝜀 × 𝑙) × (∆𝐴/∆𝑡)

where b _FeCu-DA_ is the POD active unit number of FeCu-DA (U); V is the volume of the reaction solution (μL); *ε* is the molar absorption coefficient of the colorimetric substrate, which is typically maximized at 39000 M^-1^ L cm^-1^ at 652 nm for TMB; l is the path length of light traveling in the cuvette (cm); A is the absorbance after subtraction of the blank value; and ΔA/Δt (min^-1^) is the initial rate of change in absorbance at 652 nm. The SA of the FeCu-DA (U mg^-1^) is calculated by:

SA_FeCu-DA_ = b_FeCu-DA_ / [m]

where [m] represents the mass of Fe and Cu elements (mg) contained in FeCu-DA.

*2.3 Photothermal conversion efficiency*

The photothermal conversion efficiency (*η*) of FeCu-DA can be determined on the basis of the temperature increase and decrease during a single photothermal cycle via equation (1):

$\eta=\frac{hS(Tmax-Tsurr)-Qs}{I({1-10}^{-A808})}111111111$1)

The T_max_ maximum equilibrium temperature attained during NIR irradiation. T_surr_ is the ambient environment temperature. Q_s_ is the heat associated with light absorption by the solvent, measured independently using pure water without FeCu-DA. I is the incident laser power. A_808_ is the absorbance of the samples at 808 nm. h is the heat transfer coefficient, and S is the surface area of the container. The hS was calculated from Fig. 2i according to equation 2:

$\tau_{s}=\frac{m_{D}c_{D}}{hS}$1111111112)

where τ_s_ is the sample system time constant and m_D_ and c_D_ are the mass and heat capacity of the solvent, respectively. According to the calculations, the *η* of the samples is calculated to be 46.3%.

*2.**4 ESR measurements of ·OH*

ESR measurements were conducted to detect ·OH, with DMPO serving as the spin trap to form detectable adducts. In a typical assay, FeCu-DA was used at a concentration of 20 μg mL^-1^, DMPO at 200 mM, and H_2_O_2_ at 3 mM. The components were thoroughly mixed and transferred to a quartz tube for ESR measurements for 9 minutes.

*2.5 Cell ‧OH/JC-1 detection*

The cells were cultured in a confocal culture dish at a density of 1×10^5^ cells per plate for 24 hours. The cells were subsequently incubated with fresh medium containing FeCu-DA (20 μg mL^-1^) for 12 hours. Four experimental groups were established: the Control (I), NIR (II), FeCu-DA (III), and FeCu-DA+NIR (IV) groups. In groups II and IV, the cells were exposed to an 808 nm laser (0.5 W cm^-2^) for 9 minutes. Hydroxyl radical (‧OH) levels in each group were detected via O28 staining, while changes in the mitochondrial membrane potential were assessed via JC-1 staining. Fluorescence imaging of the cells was conducted via a laser confocal microscope.

*2.6 MTT assay*

The cytotoxicity of FeCu-DA on 4T1 cells was evaluated in vitro via the MTT assay. Briefly, 5000 cells per well were seeded in a 96-well plate containing 100 μL of DMEM. After 24 hours, the cells were treated with different concentrations of FeCu-DA: 0, 5, 10, and 20 µg mL^-1^ (with NIR irradiation) or 0, 20, 50, 100, and 200 µg mL^-1^ (without NIR irradiation). For the irradiation groups, the cells were exposed to an 808 nm laser (0.5 W cm^-2^) for 9 minutes after 12 hours of incubation with FeCu-DA. The cells were then cultured for an additional 24 hours. Finally, cell viability was assessed by adding MTT solution and recording the absorbance to determine cell viability.

*2.7 3D sphere formation assay*

4T1 cells were seeded into 96-well ultralow adhesion cell culture plates at a density of 1 × 10^3^ per well and incubated for 2 days to allow the formation of tumor spheres. Tumor spheres in groups FeCu-DA and FeCu-DA+NIR were incubated with FeCu-DA (20 μg mL^-1^) for 12 hours. The tumor spheres in groups NIR and FeCu-DA+NIR were subsequently exposed to NIR irradiation (808 nm, 0.5 W cm^-2^) for 9 minutes and further cultured for 6 hours. Cell viability was evaluated via a live/dead cytotoxicity kit, and the stained cells were visualized under a fluorescence microscope.

*2.8 Cell colony formation assay*

4T1 cells were seeded into 6-well plates at a density of 300 cells per well. FeCu-DA was added to the cells according to the experimental groups, with or without NIR irradiation (808 nm, 0.5 W cm^-2^, 9 minutes). The cells were then incubated for 14 days. Following the incubation period, the colonies were fixed with 4% paraformaldehyde for 20 minutes, stained with crystal violet for an additional 20 minutes, and subsequently rinsed with PBS to remove excess dye. The number of colonies in each well was counted, and the colonies were observed under a microscope to assess the clonogenic potential of the cells.

*2.9 Cell apoptosis assay*

4T1 cells were seeded in 6-well plates and incubated for 24 hours. The cells were then subjected to one of four experimental groups, namely, the Control, NIR, FeCu-DA, or FeCu-DA+NIR groups, for an additional 24 hours. After treatment, the cells were trypsinized, collected, washed with PBS, and stained with an Annexin V-FITC/PI kit. Finally, flow cytometry was used to examine the number of apoptotic cells.

*2.10 Wound healing assay*

Cell migration was assessed via a wound-healing assay. 4T1 cells were seeded into 12-well plates and cultured for 24 hours to form a confluent monolayer. A sterile pipette tip was used to scratch the cell monolayer, creating a wound. Cellular debris was removed by gently washing with PBS. The cells were then incubated with FeCu-DA for 12 hours, followed by NIR irradiation as required for the experimental groups. After irradiation, the cells were cultured in fresh medium for an additional 48 hours. The wound region was photographed at 0 and 48 h under a microscope. Cell migration toward the wound site was quantified by determining wound closure via ImageJ software.

*2.1**1 Immunofluorescence staining*

The cells were subjected to one of four experimental groups: the Control, NIR, FeCu-DA, or FeCu-DA+NIR groups. The treated cells were subsequently fixed with 4% paraformaldehyde, permeabilized with 0.5% Triton X-100, and blocked with QuickBlock™ Blocking Buffer. Afterwards, the cells were incubated overnight at 4°C with anti-Bax, anti-Bcl-2, anti-CRT and anti-HMGB1 antibodies, followed by incubation with secondary antibodies (Alexa Fluor 488-labeled goat anti-rabbit IgG or Cy3-labeled goat anti-rabbit IgG) and counterstaining with DAPI. Immunofluorescence micrographs were captured via confocal laser scanning microscopy to analyze protein localization and expression.

*2.12 Western blot*

Western blot analysis was conducted to assess protein expression after the four experimental groups were treated. First, proteins were extracted from treated 4T1 cells. The protein samples were then subjected to sodium dodecyl sulfate‒polyacrylamide gel electrophoresis (SDS‒PAGE) and transferred onto PVDF membranes. After being washed and blocked, the obtained PVDF membranes were incubated overnight at 4°C with different primary antibodies. Next, the membranes were incubated with anti-rabbit or anti-mouse HRP-conjugated polyclonal secondary antibodies. Protein bands were detected via enhanced chemiluminescence (ECL) reagents and subsequently scanned. Band intensities were quantified via ImageJ software.

*2.13 ATP assay*

An ATP assay was performed to evaluate the extracellular release of ATP. First, 1x10^4^ 4T1 cells per well were seeded in a 24-well plate and incubated for 24 hours. Then, the cells were incubated with FeCu-DA (20 μg mL^-1^), with or without subsequent NIR irradiation. After the treatment, the extracellular ATP levels were quantified via an ATP assay kit according to the manufacturer's instructions.

**Supplementary Figures**


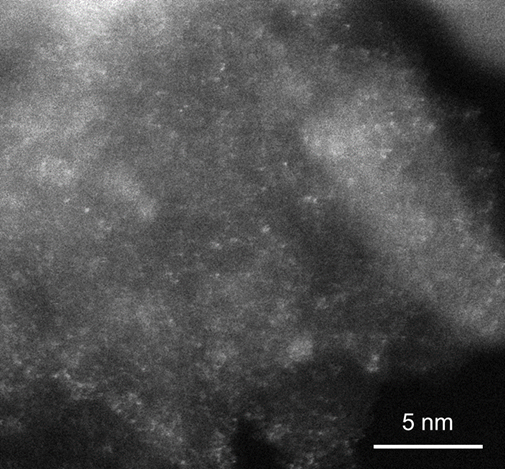


**Figure S1.** HAADF-STEM image of Fe-SA.


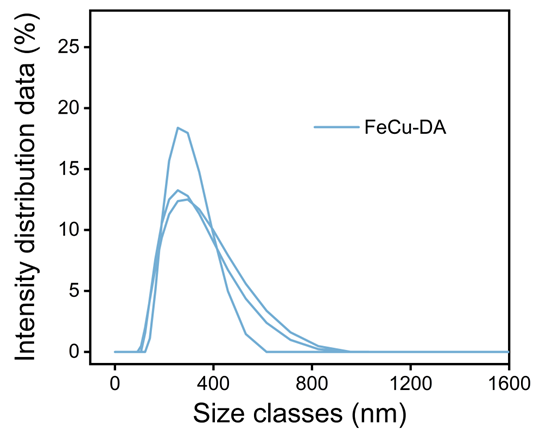


**Figure S2.** The dynamic light scattering (DLS) data of FeCu-DA.


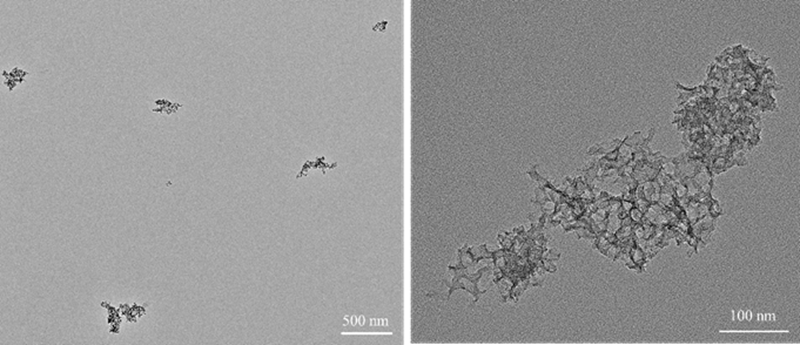


**Figure S3.** Low-magnification TEM of FeCu-DA.


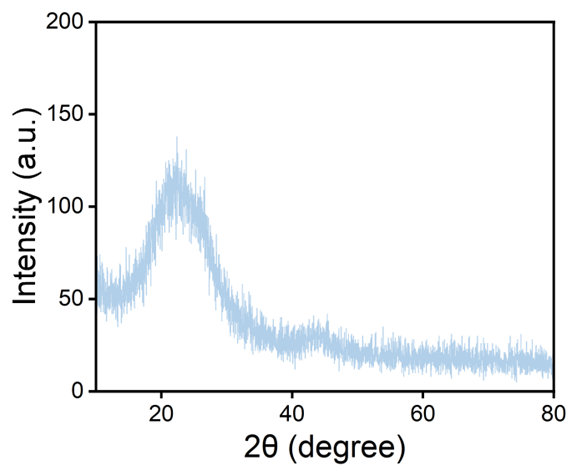


**Figure S4.** XRD pattern of FeCu-DA.


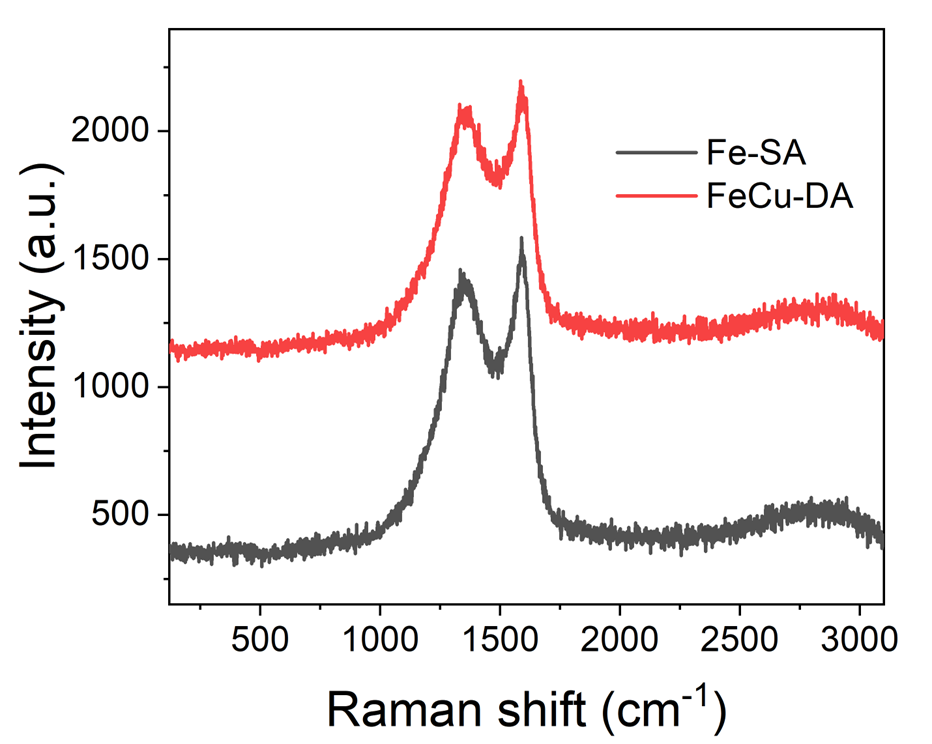


**Figure S5**. Raman spectra of Fe-SA and FeCu-DA.


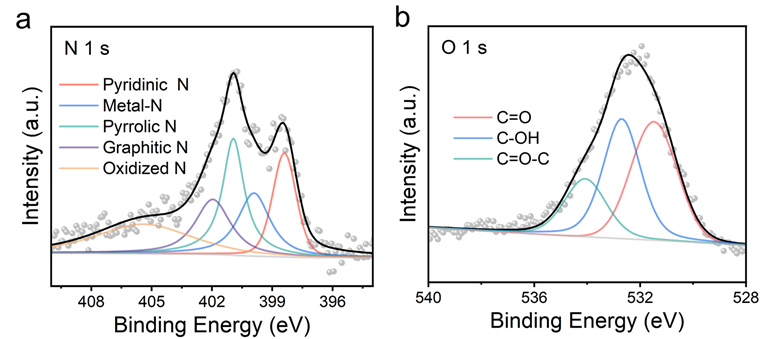


**Figure S6.** High resolution N 1 s (a) and O 1 s (b) XPS spectra of FeCu-DA.


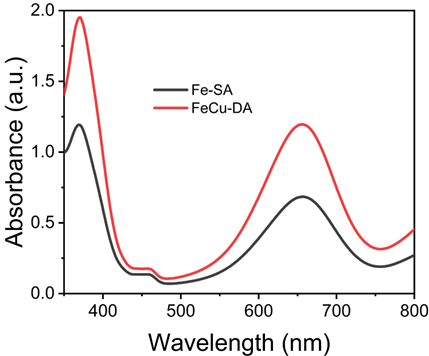


**Figure S7.** POD activity of Fe-SA and FeCu-DA.


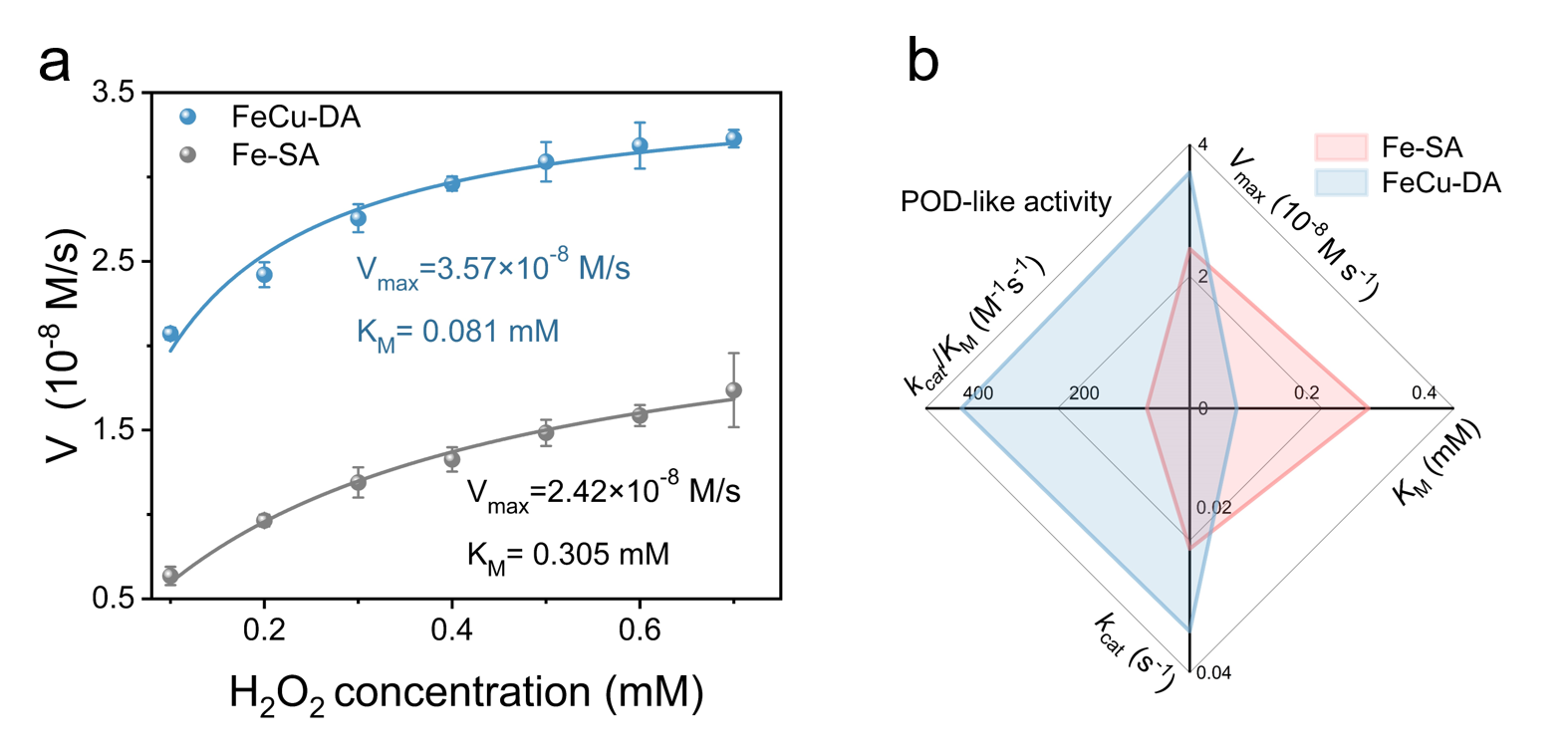


**Figure S8**. a) Michaelis‒Menten curves for FeCu-DA and Fe-SA. b) The kinetics parameters of FeCu-DA and Fe-SA. H_2_O_2_ is the substrate.


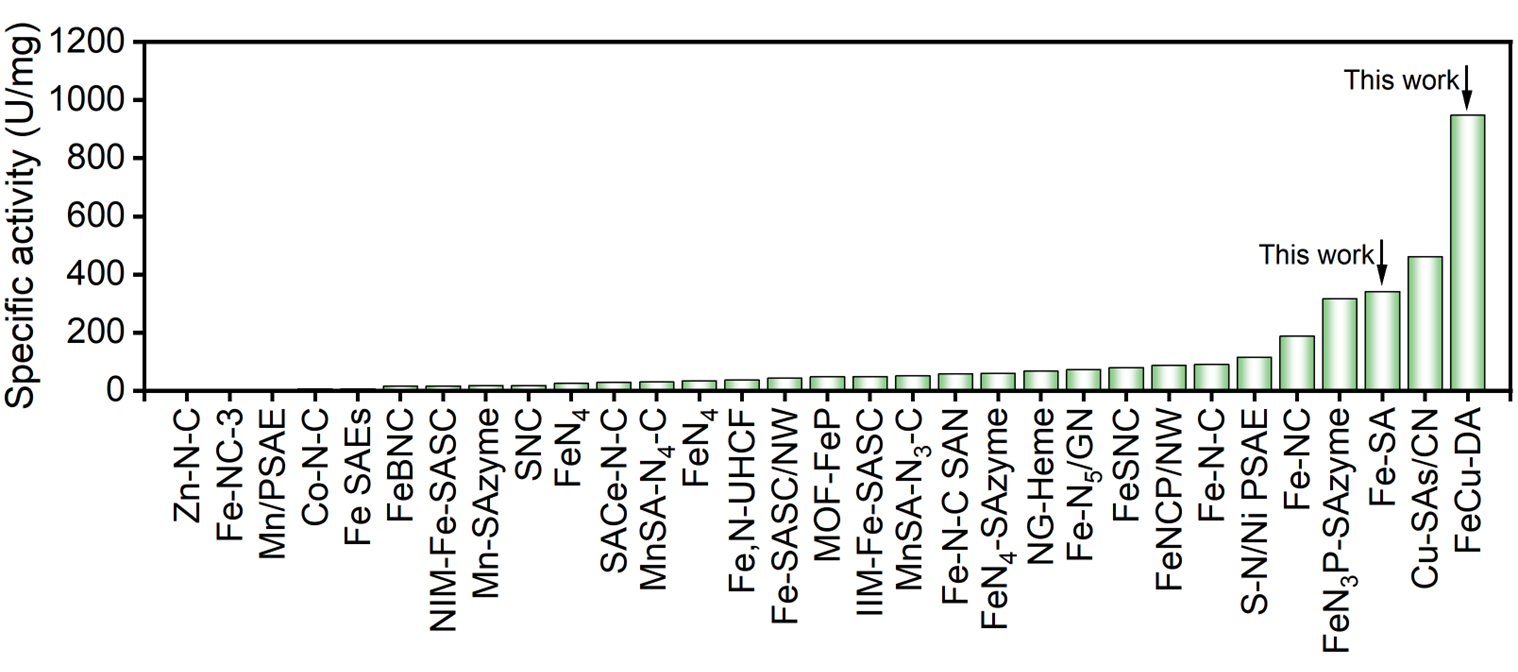


**Figure S9.** Comparison of the specific activities of FeCu-DA with those of previously reported non-noble metal SAzymes.


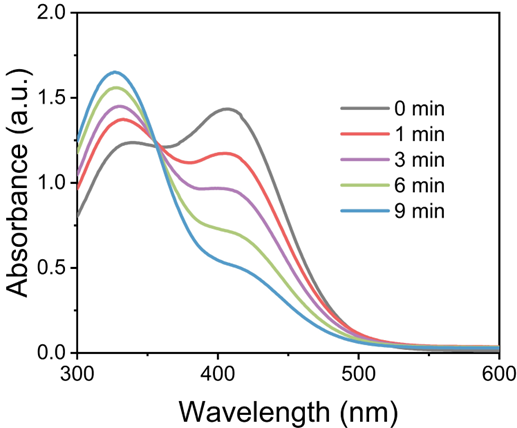


**Figure S10.** GSH-OXD activities at different times.


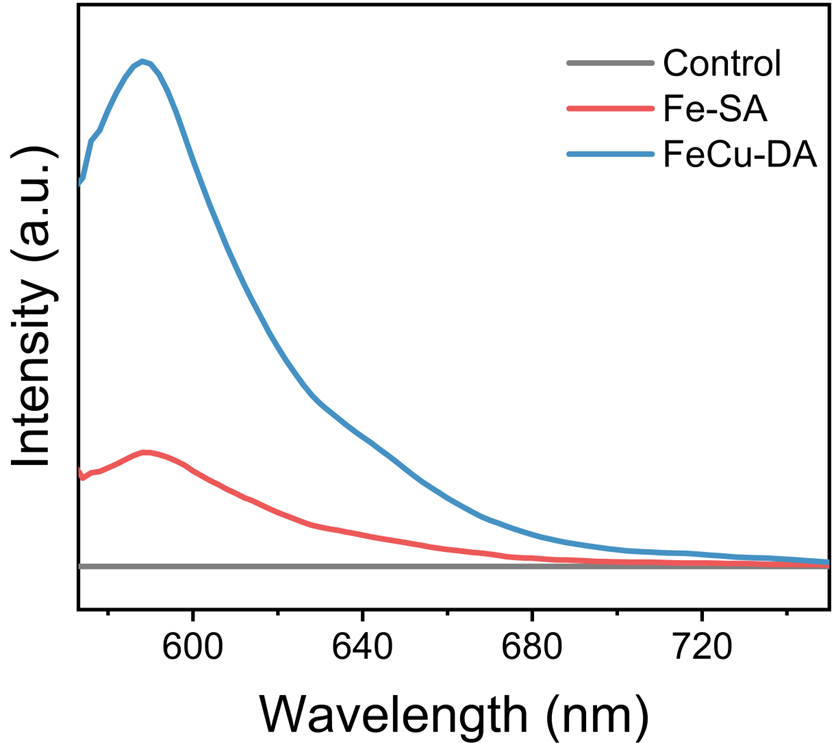


**Figure S11.** Detection of H_2_O_2_ production. The concentrations of FeCu-DA and Fe-SA were 100 µg mL^-1^. The concentration of GSH was 2 mM. The reaction time was 2 hours.


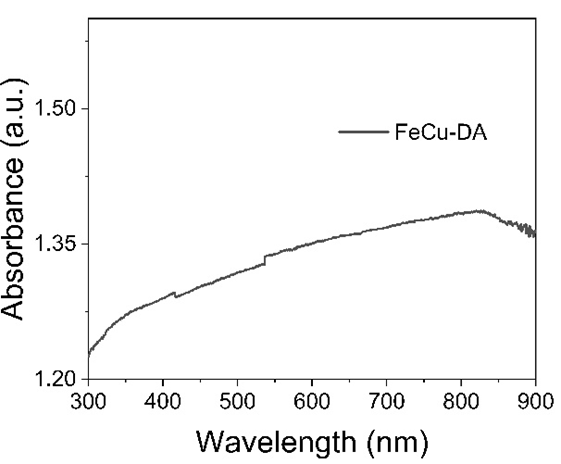


**Figure S12.** UV/Vis-NIR absorption spectrum of FeCu-DA.


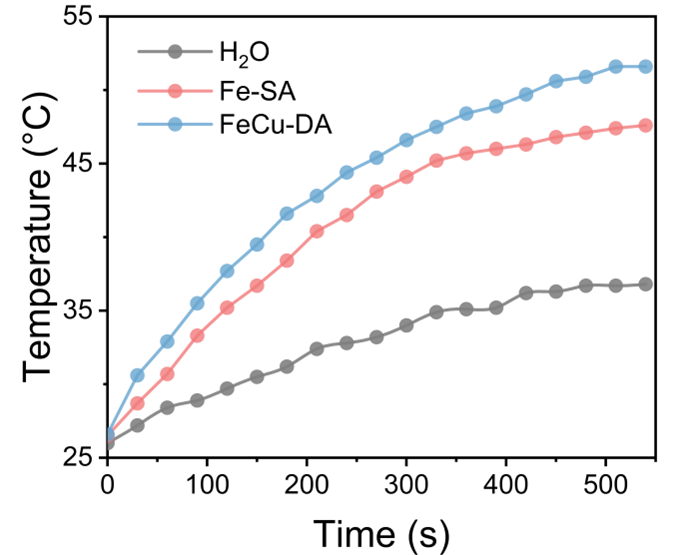


**Figure S13.** Temperature elevation curves of FeCu-DA and Fe-SA under 808 nm NIR irradiation.


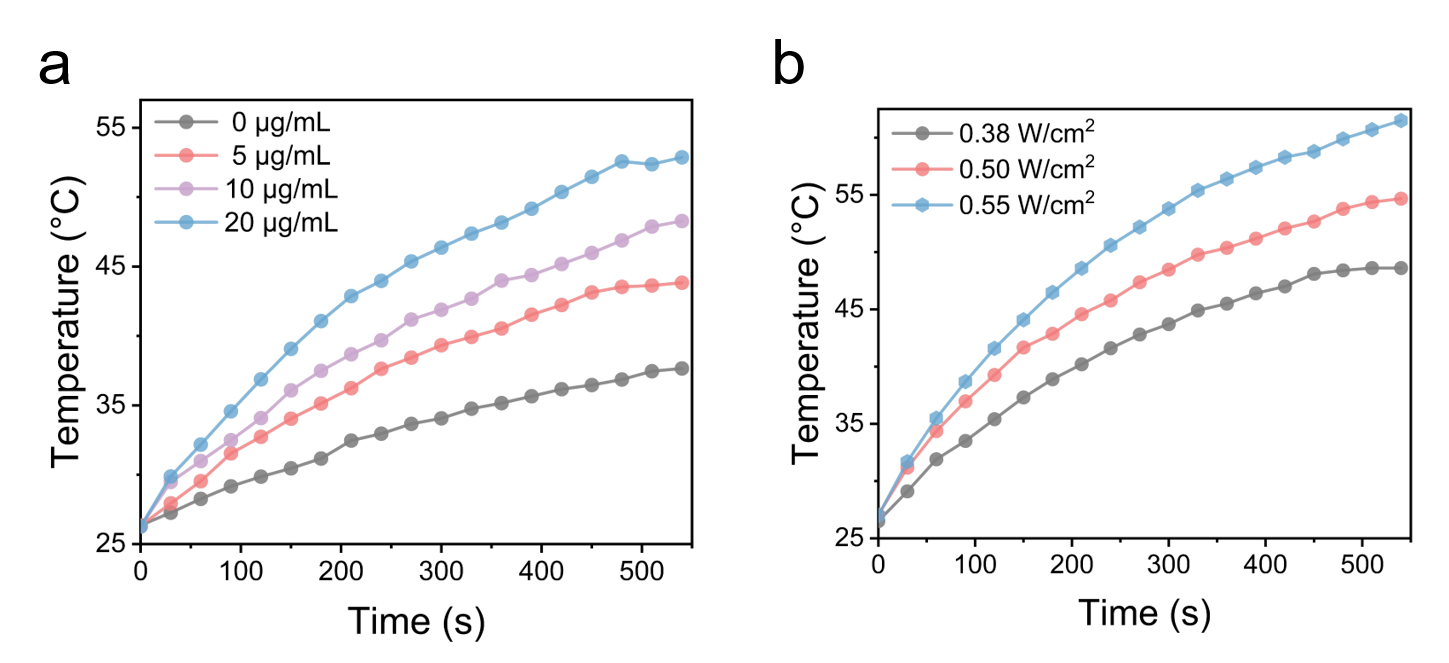


**Figure S14.** Temperature elevation curves of FeCu-DA solutions a) at different concentrations and b) under 808 nm NIR irradiation at different power densities.


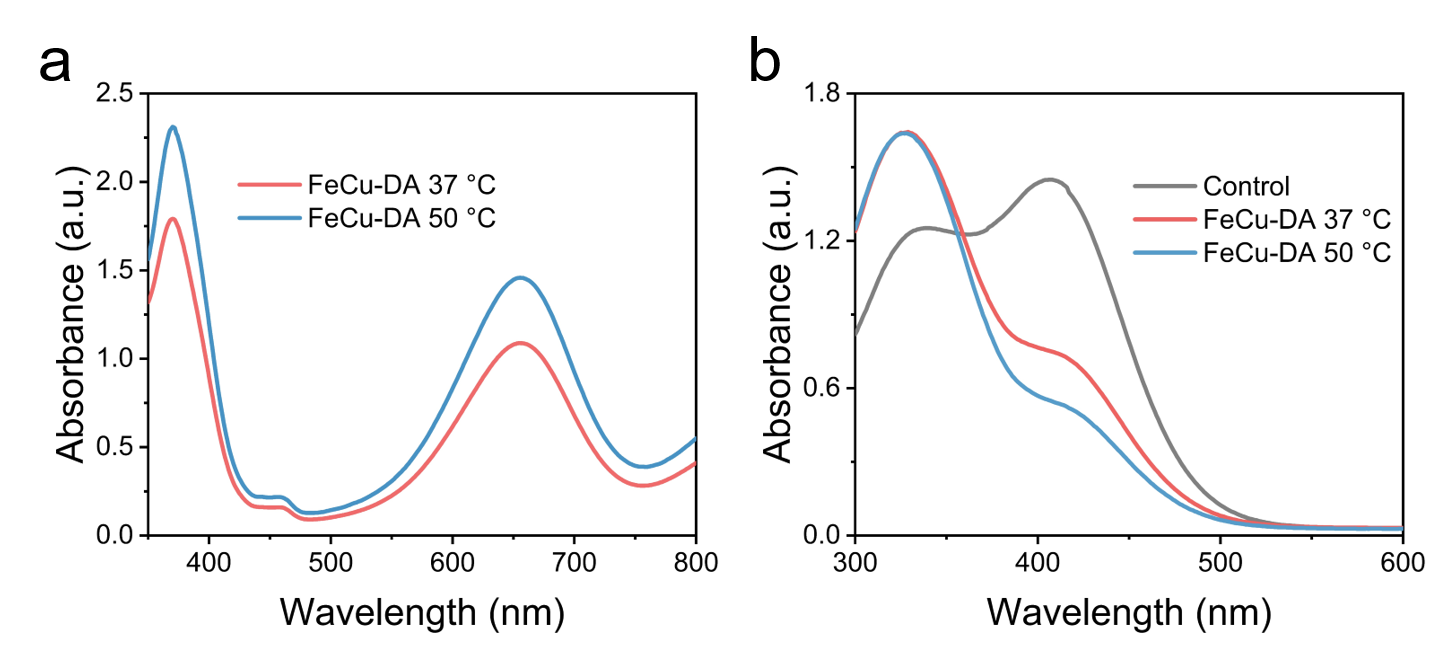


**Figure S15.** Comparison of a) POD and b) GSH-OXD activities of FeCu-DA at different temperatures.


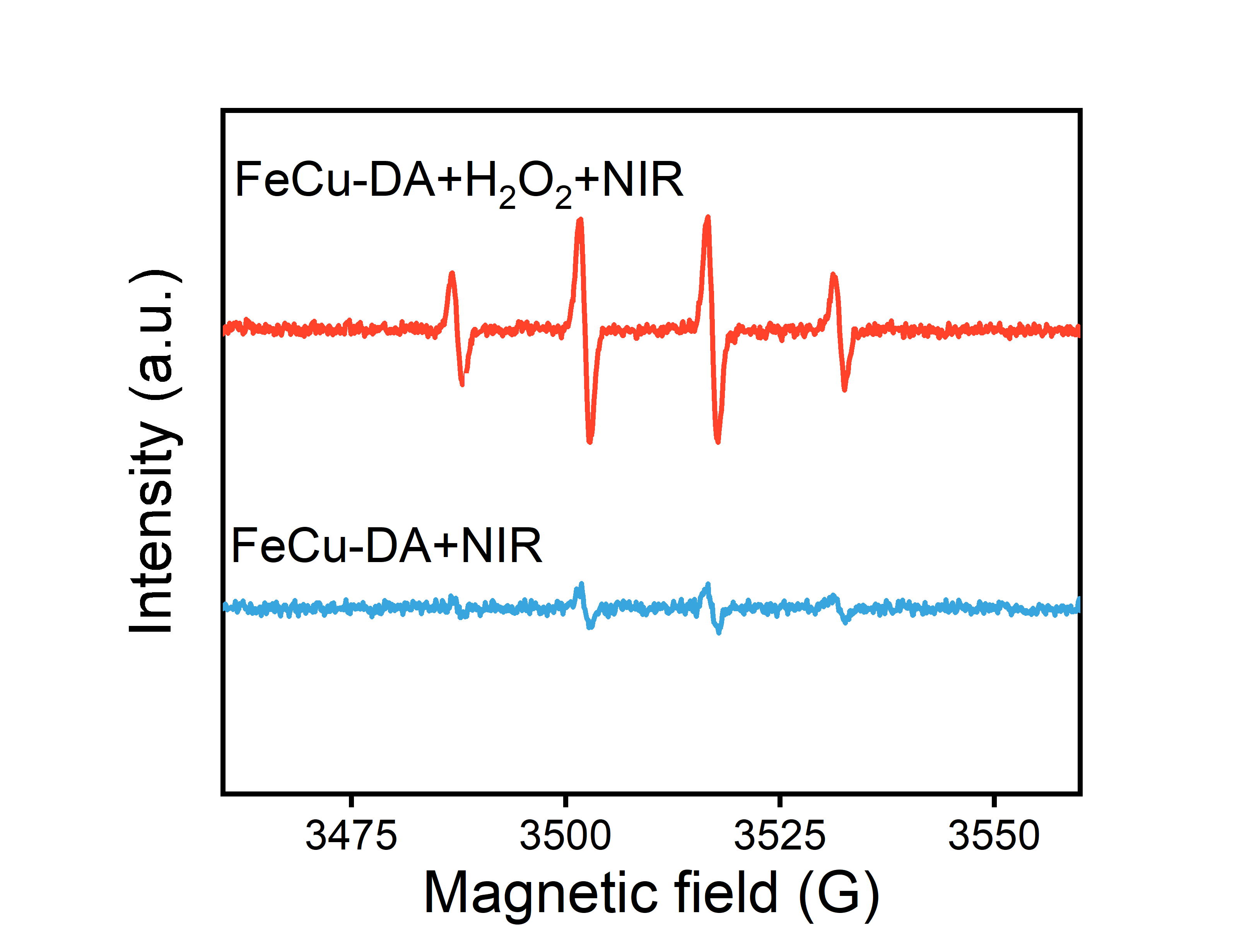


**Figure S16.** ESR spectra of DMPO-OH of FeCu-DA under NIR irradiation.


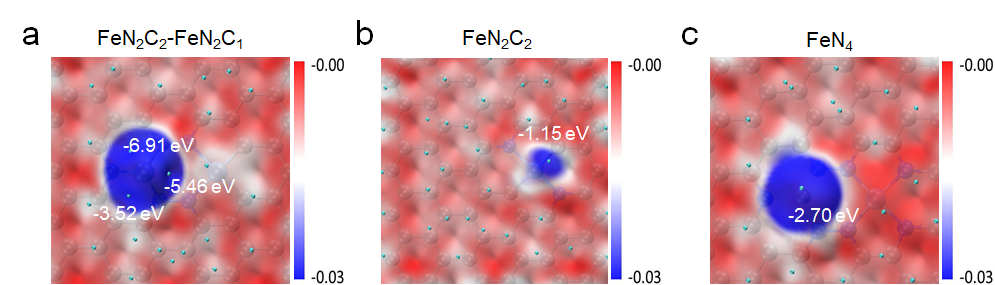


**Figure S****17.** Local electron attachment energy (LEAE) of a) FeFe-DA with the FeN_2_C_2_-FeN_2_C_1_ configuration, b) Fe-SA with the FeN_2_C_2_ configuration and c) Fe-SA with the FeN_4_ configuration.


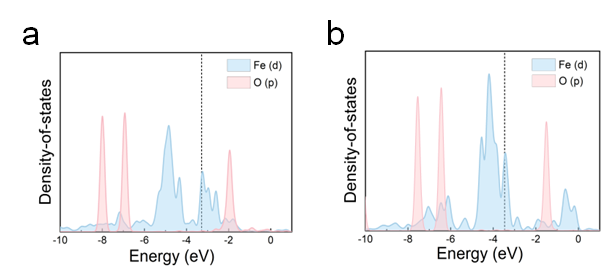


**Figure S****18.** a) PDOS of the H_2_O_2_/FeFe-DA surface with the FeN_2_C_2_-FeN_2_C_1_ configuration. b) PDOS of H_2_O_2_/Fe-SA with the FeN_2_C_2_ configuration.


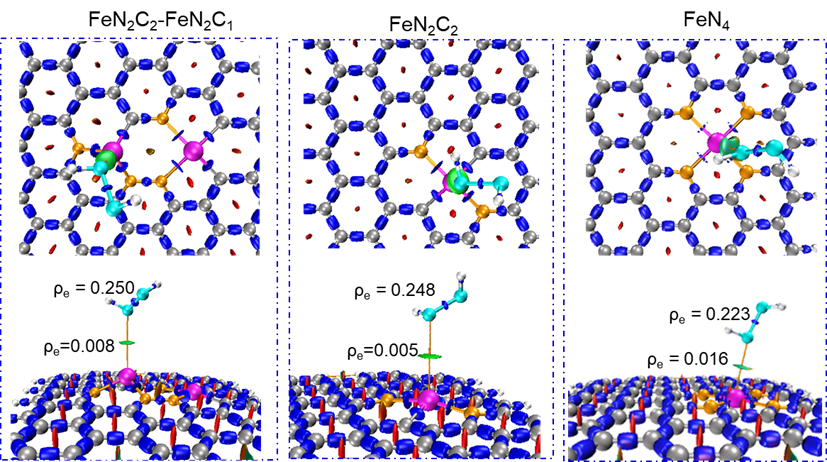


**Figure S19.** Interaction region indicator and electron density of selected bond critical points for the H_2_O_2_/FeFe-DA and Fe-SA surfaces.


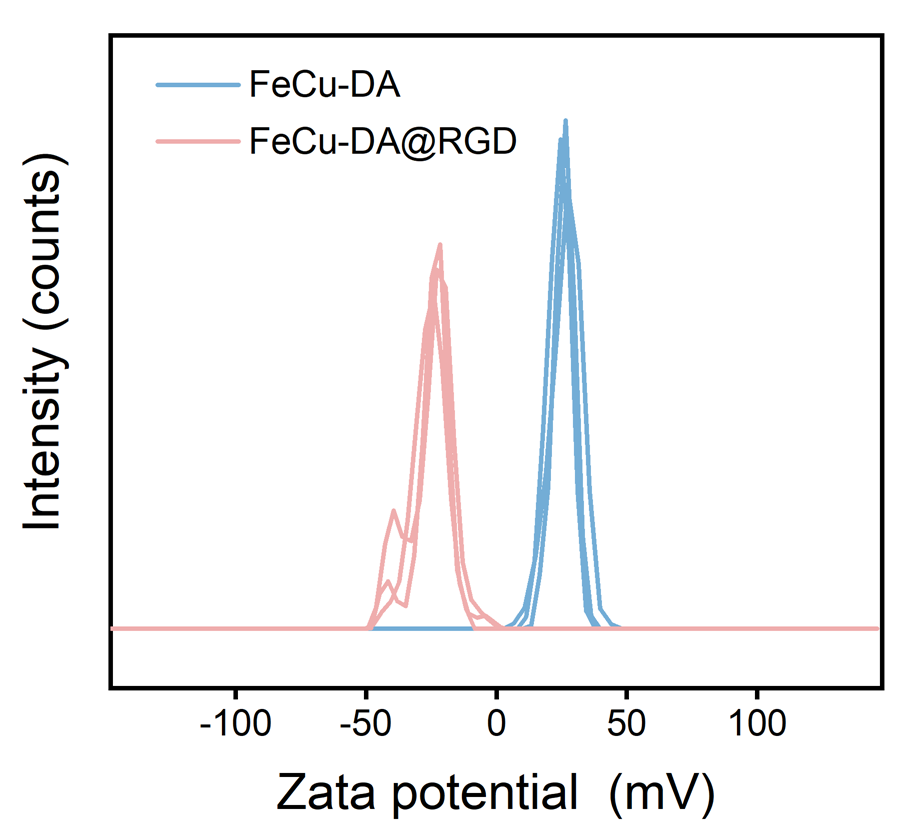


**Figure S20.** Zeta potential of FeCu-DA and FeCu-DA@RGD.


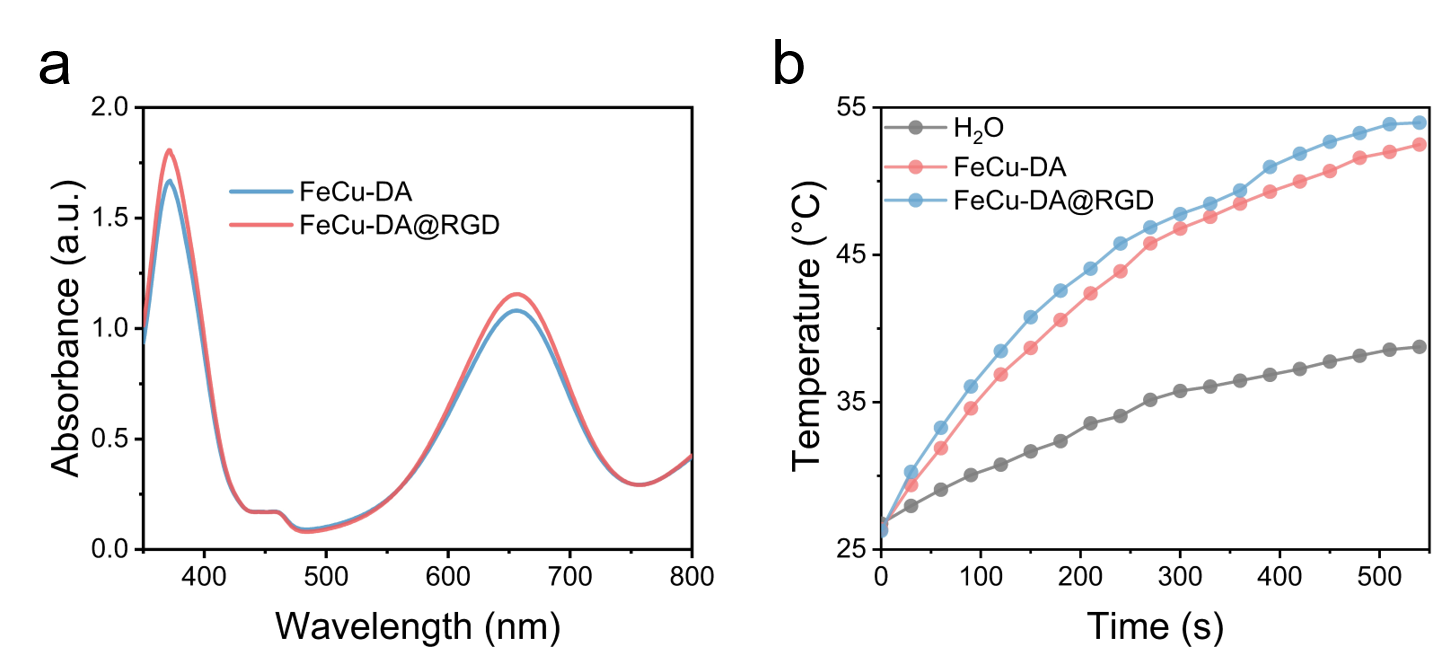


**Figure S****21.** Comparison of a) POD activity and b) photothermal effects of FeCu-DA and FeCu-DA@RGD. The laser power density was 0.5 W cm^-2^ and the catalyst concentration was 20 μg mL^-1^.


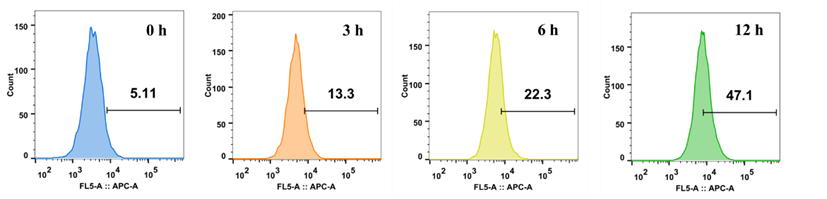


**Figure S22.** Flow cytometry assay of 4T1 cells treated with Cy5.5-labeled FeCu-DA.


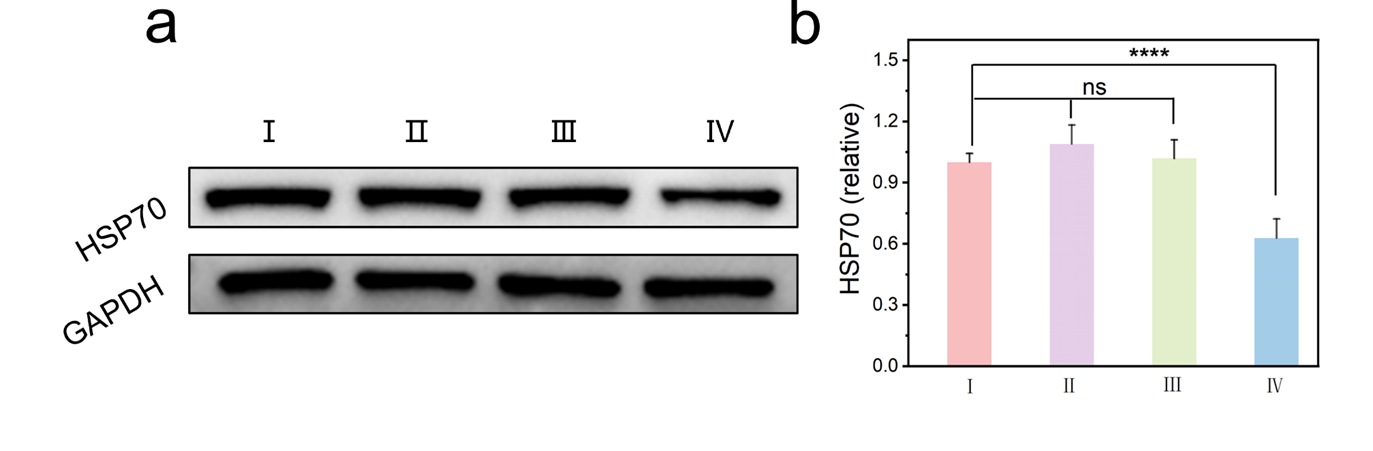


**Figure S23.** a) Western blot analysis of the expression of HSP70 in 4T1 cells after various treatments. b) Analysis of the gray value of HSP70 in Figure a. Treatment group Ⅰ: Control; group Ⅱ: NIR; group Ⅲ: FeCu-DA; and group Ⅳ: FeCu-DA+NIR. All the quantitative data are presented as the means ± SDs (n = 3). P values were calculated via one-way ANOVA: *P < 0.05, **P < 0.01, ***P < 0.001, ****P < 0.0001, and no significant difference (ns).


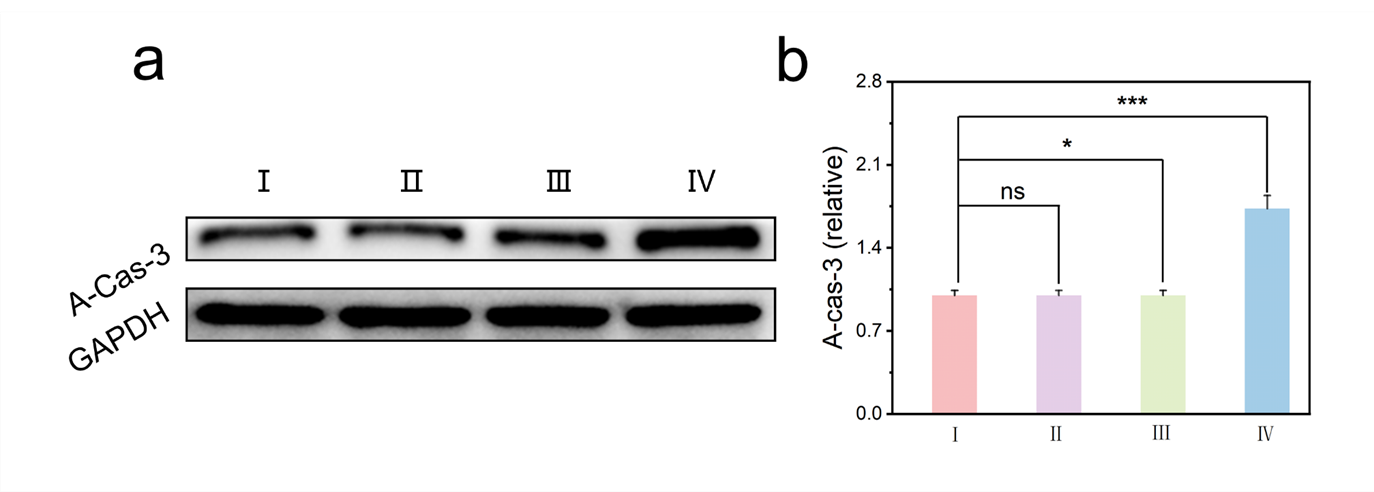


**Figure S****24.** a) Western blot analysis of the protein expression of caspase-3 in 4T1 cells after various treatments. b) Analysis of the gray value of caspase-3 in Figure a. Treatment group Ⅰ: Control; group Ⅱ: NIR; group Ⅲ: FeCu-DA; and group Ⅳ: FeCu-DA+NIR. All the quantitative data are presented as the means ± SDs (n = 3). P values were calculated via one-way ANOVA: *P < 0.05, **P < 0.01, ***P < 0.001, ****P < 0.0001, and no significant difference (ns).


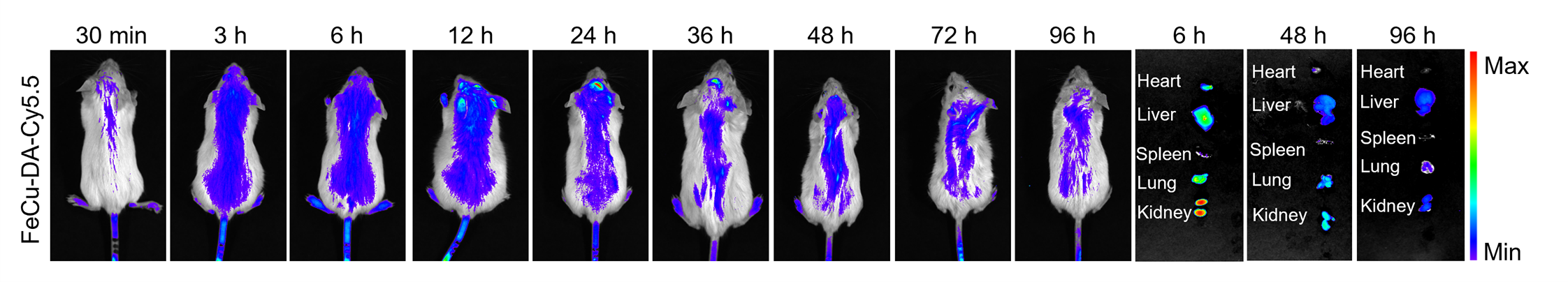


**Figure S25**. Time-dependent in vivo biodistribution of FeCu-DA-Cy5.5 in healthy mice, along with its biodistribution in major organs at specific time points postinjection.


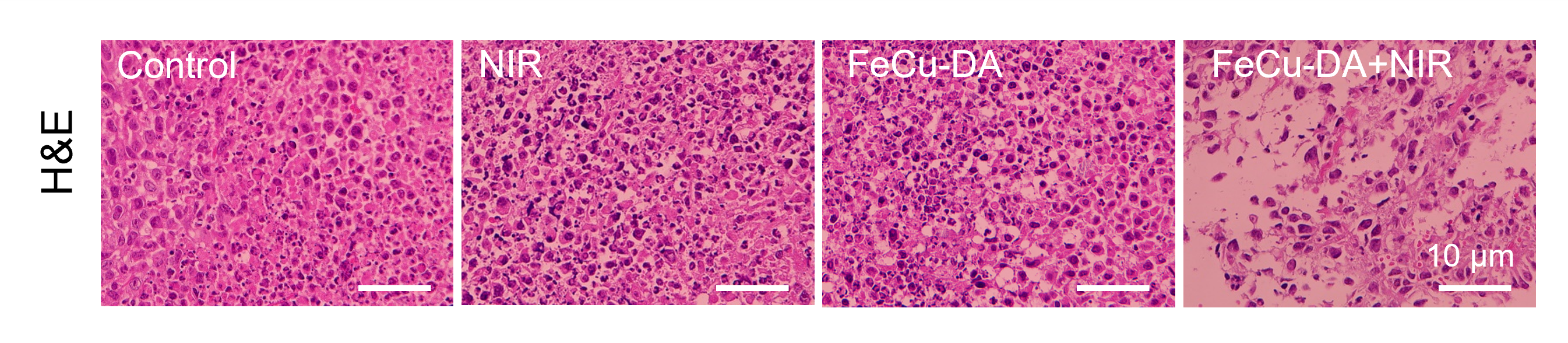


**Figure S26.** H&E staining of tumor tissues.


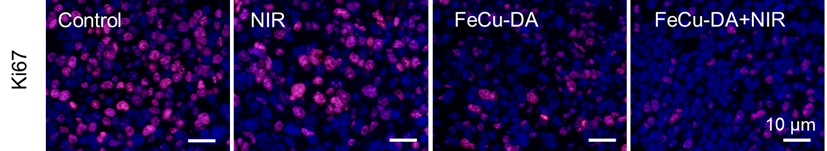


**Figure S27.** Ki67 staining of tumor tissues.


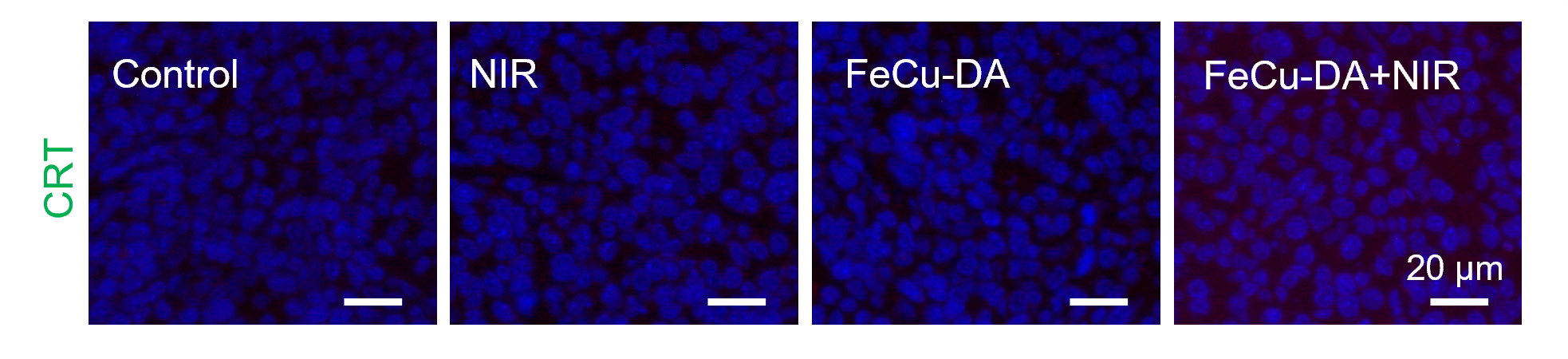


**Figure S28.** Immunofluorescence images of tumor slices with nuclei stained by DAPI, and CRT stained by its antibody.


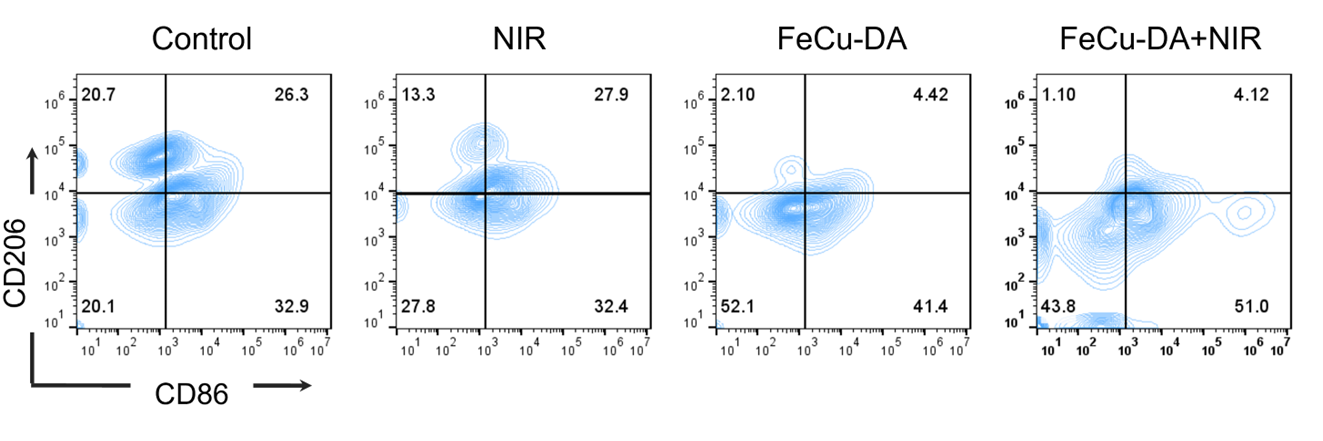


**Figure S29.** Representative flow cytometric plots of CD86^+^ CD206^+^ T-cell populations in the spleen after various treatments.


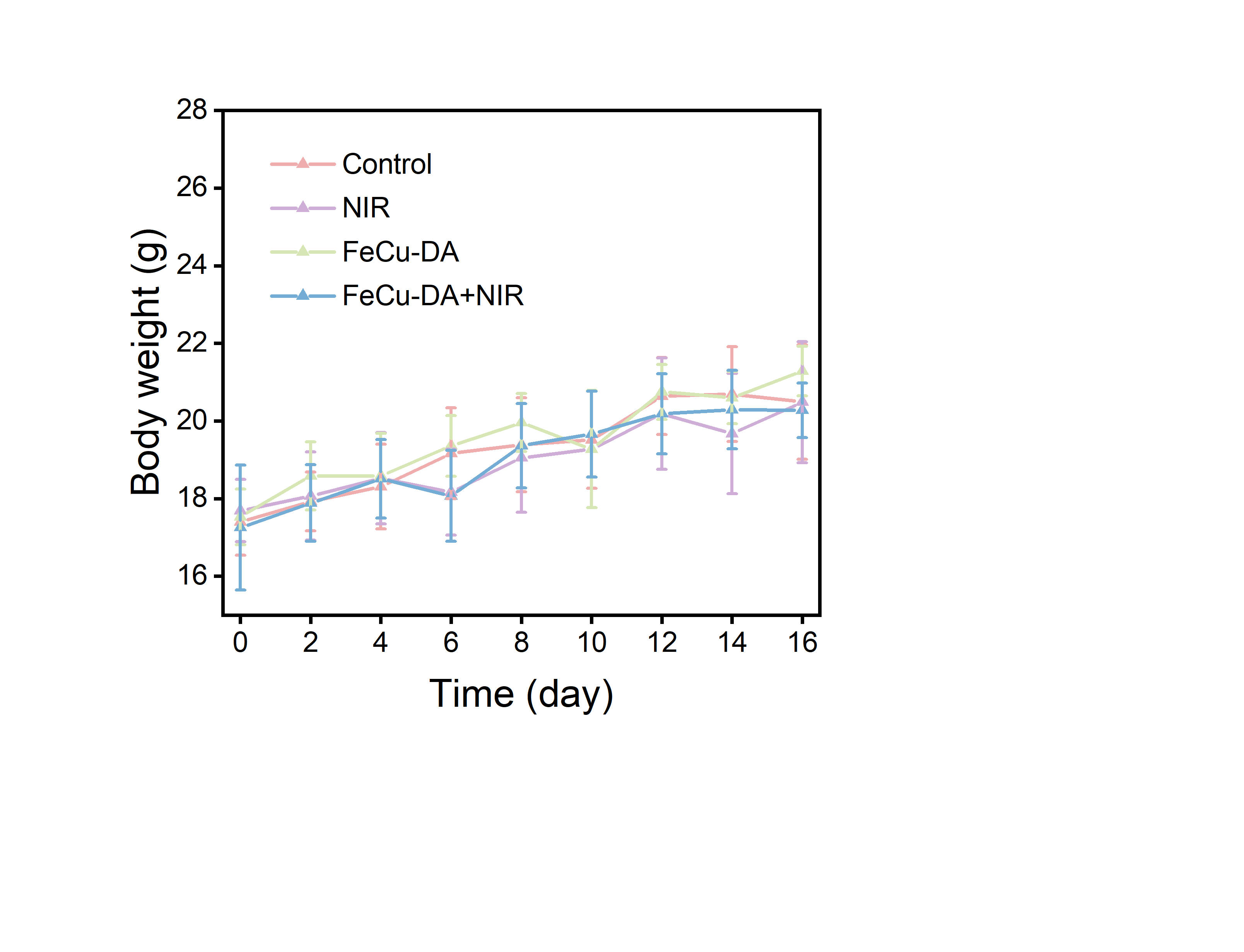


**Figure** **S30.** Changes in body weight during treatments (n = 5).


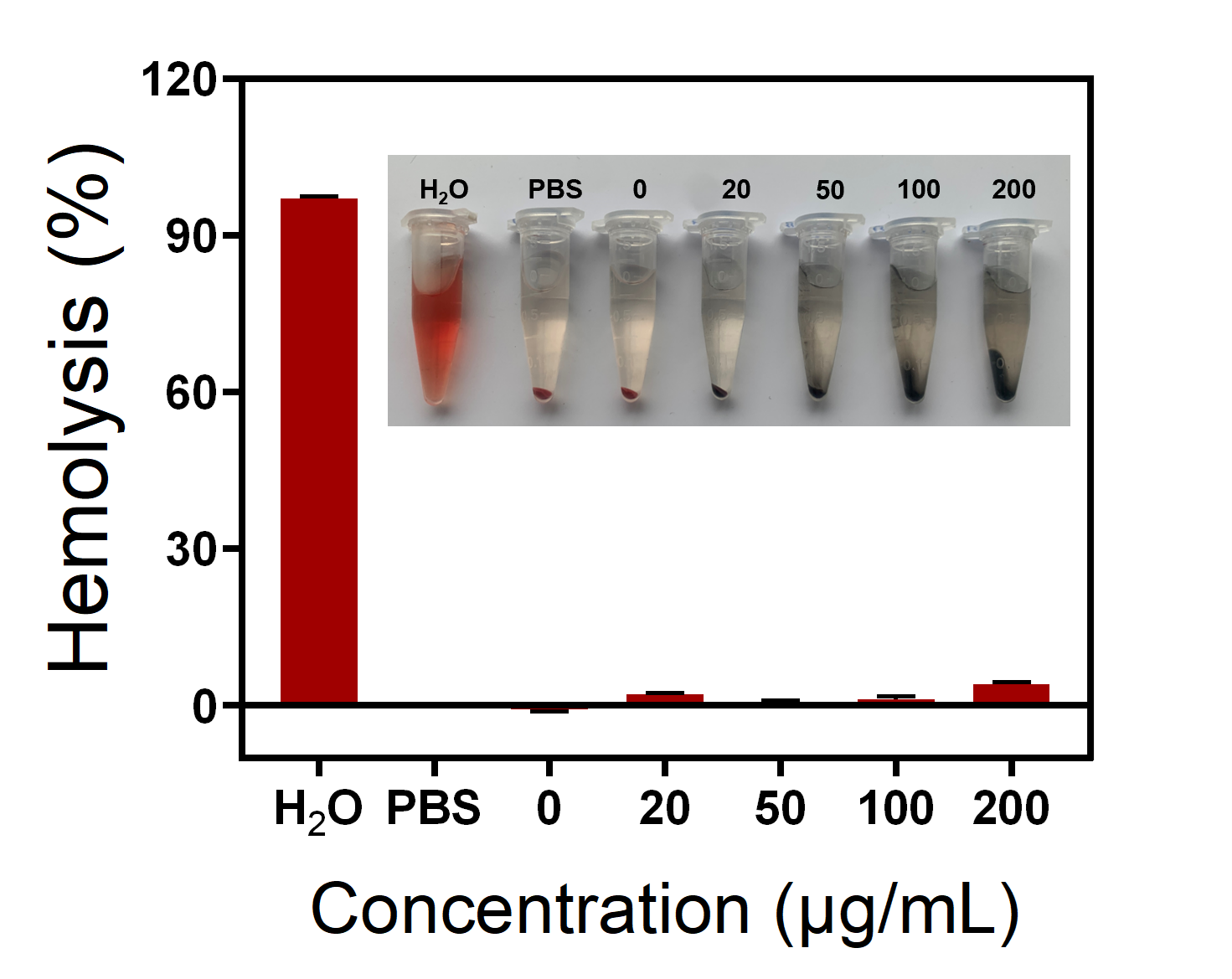


**Figure S31.** Hemolysis assay of FeCu-DA (n=3).


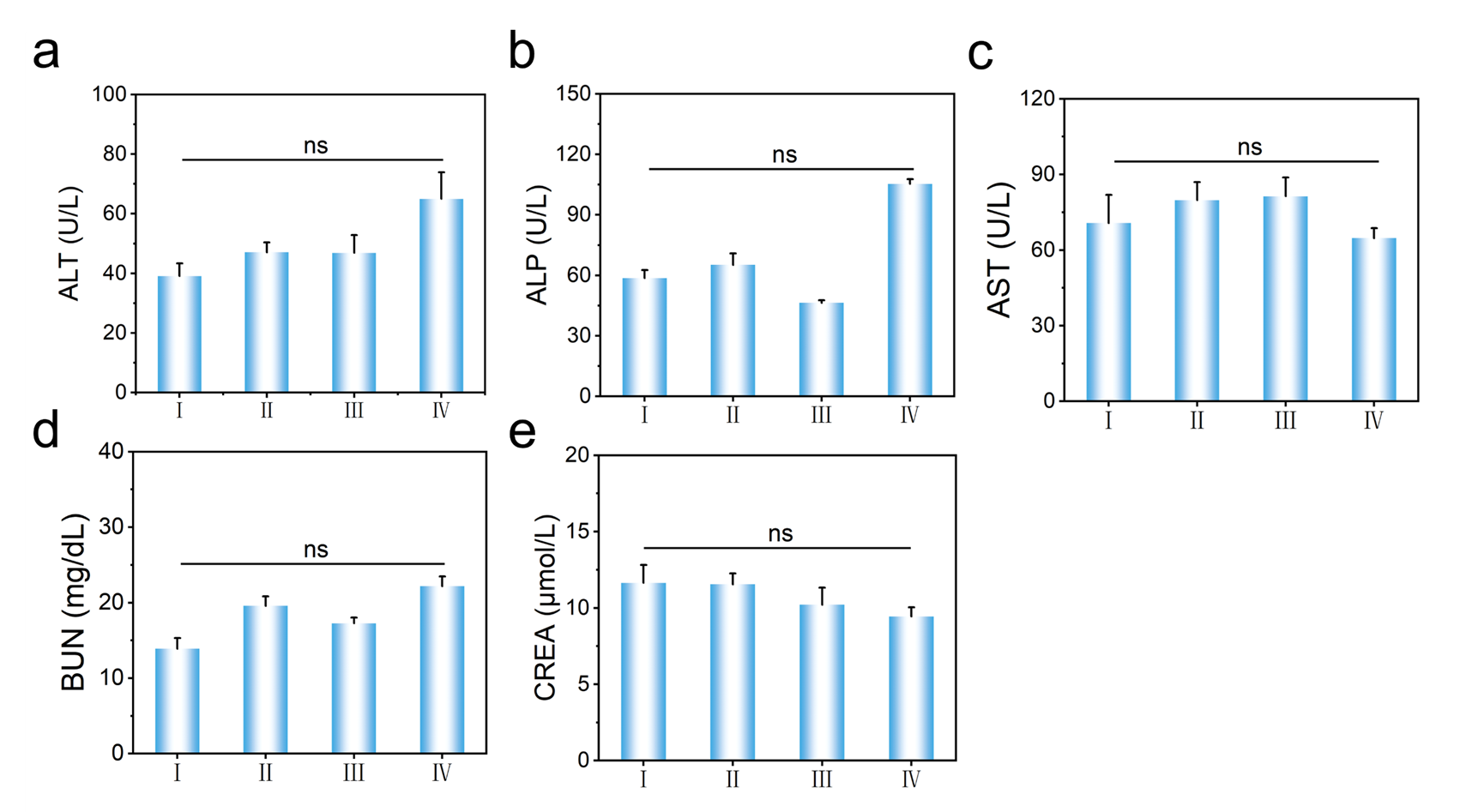


**Figure S****32.** Hematology analysis of treated mice. The terms include a) alanine aminotransferase (ALT), b) alkaline phosphatase (ALP), c) aspartate aminotransferase (AST), d) blood urea nitrogen (BUN) and e) creatinine (CREA). Treatment group Ⅰ: Control; group Ⅱ: NIR; group Ⅲ: FeCu-DA; and group Ⅳ: FeCu-DA+NIR. All the quantitative data are presented as the means ± SDs (n = 4). P values were calculated via one-way ANOVA: *P < 0.05, **P < 0.01, ***P < 0.001, ****P < 0.0001, and no significant difference (ns).


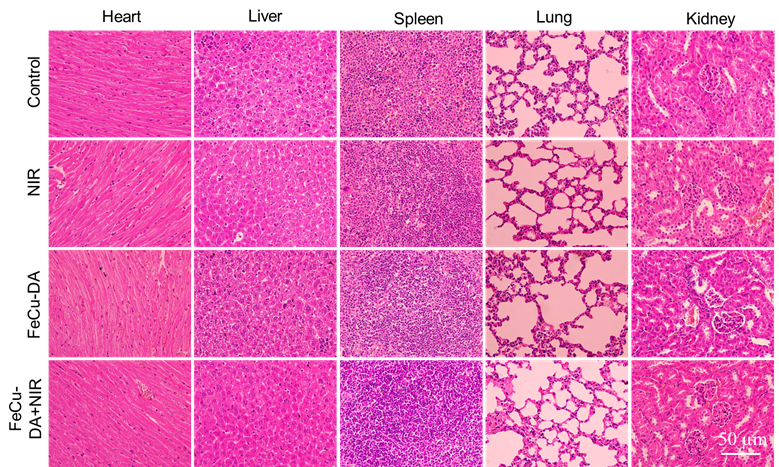


**Figure S33.** H&E-stained tissue sections of major organs (heart, liver, spleen, lung, and kidney) from mice in different treatment groups.


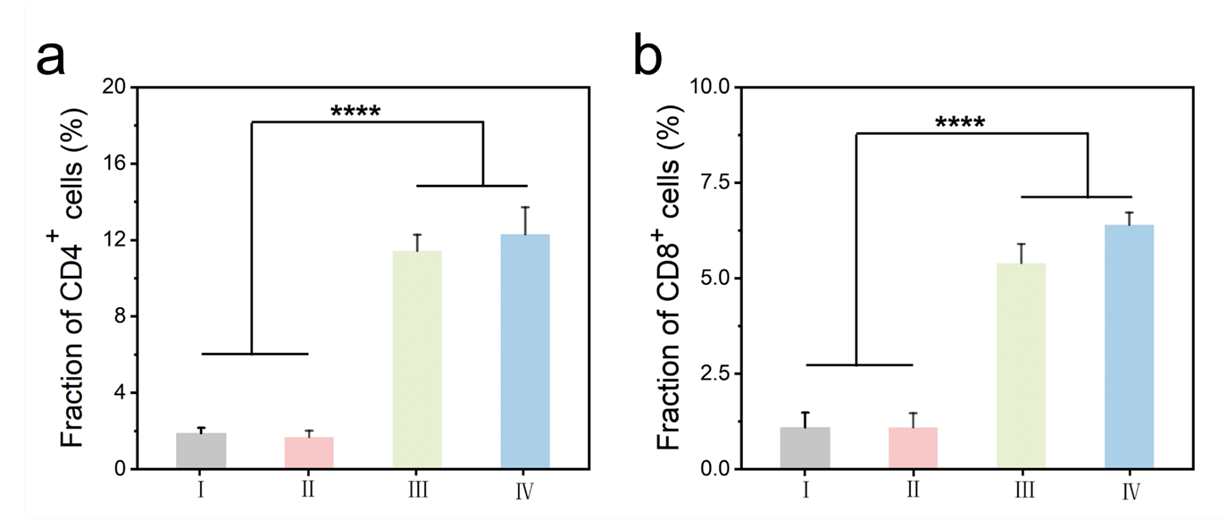


**Figure S****34.** Semiquantitative analysis of CD4^+^ and CD8^+^ expression is shown in Figure 7g. Treatment group Ⅰ: Control; group Ⅱ: αPD-L1; group Ⅲ: FeCu-DA+NIR; and group Ⅳ: FeCu-DA+NIR+αPD-L1. All the quantitative data are presented as the means ± SDs (n = 3), and P values were calculated via one-way ANOVA: *P < 0.05, **P < 0.01, ***P < 0.001, ****P < 0.0001, and no significant difference (ns).

**Supplementary Tables**

**Table S1** Structural parameters of FeCu-DA extracted from the EXAFS fitting.

| **Sample** | **Shell** | **CN** | **R (Å)** | **σ^2^ (10^−2^ Å^2^)** | **ΔE_0_ (eV)** | **r-factor (%)** |
| --- | --- | --- | --- | --- | --- | --- |
| Cu Foil | Cu-Cu | - | - | - | - | - |
| Sample | Cu-N/C | 2.0 | 1.95 | 0.3 | -4.6 | 0.4 |
|  | Cu-N/C | 0.9 | 2.11 | 0.3 | -4.6 |  |
| Fe Foil | Fe-Fe | - | - | - | - | - |
| Sample | Fe-N/C | 3.8 | 2.00 | 1.0 | -8.0 | 0.4 |
|  | Fe-N/C | 2.0 | 2.58 | 0.4 | -5.3 |  |

^a^CN is the coordination number for the absorber–backscatterer pair, R is the average absorber−backscatterer distance, σ^2^ is the Debye−Waller factor, and ΔE_0_ is the inner potential correction.

The data ranges used for data ﬁtting in k-space (Δk) and R-space (ΔR) are 3.5–10.5 Å^−1^ and 1.0–2.5 Å, respectively.

The S_0_^2^ values for the Cu and Fe K-edge EXAFS fits are determined to be 0.8 and 1.0, respectively.

**Table S2** Comparison of POD kinetic parameters of FeCu-DA and Fe-SA.

| **Materials** | **Substrate** | **[E/Metal]**  **(M)** | **K_M_ (mM)** | **V_max_**  **(M s^-1^)** | **k_cat_**  **(s^-1^)** | **k_cat_/K_M_**  **(M^-1^s^-1^)** |
| --- | --- | --- | --- | --- | --- | --- |
| Fe-SA | TMB | 1.90×10^-7^ | 0.74 | 3.65×10^-8^ | 0.19 | 256.8 |
| FeCu-DA |  | 2.06×10^-7^ | 0.21 | 6.42×10^-8^ | 0.31 | 1476.2 |
| Fe-SA | H_2_O_2_ | 9.49×10^-7^ | 0.305 | 2.42×10^-8^ | 0.025 | 82.0 |
| FeCu-DA |  | 1.03×10^-6^ | 0.081 | 3.57×10^-8^ | 0.035 | 432.1 |

**Table S3** Comparison of the specific activities of FeCu-DA with those of Fe-SA and previously reported non-noble metal SAzymes.

| **SAzymes** | **Specific activity (U mg^-1^)** | **References** |
| --- | --- | --- |
| **Fe-SA** | **340.93** | **This work** |
| **FeCu-DA** | **948.05** | **This work** |
| Zn-N-C | 2.46 | [S6] |
| Fe-NC-3 | 3 | [S7] |
| Mn/PSAE | 5.04 | [S8] |
| Co-N-C | 6.33 | [S6] |
| Fe SAEs | 6.75 | [S9] |
| FeBNC | 15.41 | [S10] |
| NIM-Fe-SASC | 16.6 | [S11] |
| Mn-SAzyme | 17.48 | [S12] |
| SNC | 17.5 | [S13] |
| FeN_4_ | 25.33 | [S6] |
| SACe-N-C | 29.61 | [S14] |
| Mn_SA_-N_4_-C | 29.9 | [S15] |
| FeN_4_ | 33.8 | [S16] |
| Fe,N-UHCF | 36.6 | [S17] |
| Fe-SASC/NW | 42.8 | [S18] |
| MOF-FeP | 48.07 | [S19] |
| IIM-Fe-SASC | 48.5 | [S11] |
| Mn_SA_-N_3_-C | 51.3 | [S15] |
| Fe-N-C SAN | 57.76 | [S20] |
| FeN_4_-SAzyme | 60.51 | [S21] |
| NG-Heme | 67.3 | [S22] |
| Fe-N_5_/GN | 72 | [S23] |
| FeSNC | 79.71 | [S24] |
| FeNCP/NW | 86.9 | [S25] |
| Fe-N-C | 90.11 | [S26] |
| S‒N/Ni PSAE | 115 | [S27] |
| Fe-NC SAC | 188 | [S28] |
| FeN_3_P-SAzyme | 316 | [S16] |
| Cu-SAs/CN | 461.6 | [S29] |

**Table S4** Comparison of the photothermal conversion efficiency (PCE) for FeCu-DA and other nanozymes.

| **Nanozyme** | **PCE (*η*)** | **Wavelength** | **Laser power**  **(W cm^-2^)** | **References** |
| --- | --- | --- | --- | --- |
| **FeCu-DA** | **46.3%** | **808 nm** | **0.5** | **This work** |
| Au-FeSAzyme | 41.2% | 808 nm | 0.5 | [S30] |
| FeSAzyme | 32% | 808 nm | 0.5 | [S30] |
| Cu_7_S_4_ NCs | 25.4% | 808 nm | 0.5 | [S31] |
| Cu-JMCNs | 42.1% | 980 nm | 1.0 | [S32] |
| Cu SAzyme | 41.6% | 1064 nm | 0.63 | [S33] |
| Cu SAzyme | 37.2% | 808 nm | 0.63 | [S33] |
| Fe-SAzyme | 41.2% | 1064 nm | 0.5 | [S34] |
| FeSA-HNCSB | 42% | 808 nm | 1.0 | [S35] |
| Fe-N/C | 16.86% | 808 nm | 3.0 | [S36] |
| GNPs | 37% | 808 nm | 1.0 | [S37] |
| PmMn/SAE | 22.1% | 1064 nm | 1.2 | [S38] |
| PBSA | 31.98% | 808 nm | 1.0 | [S39] |
| Pd - SAzyme | 33.98% | 1064 nm | 0.69 | [S40] |
| P-MOF | 41% | 808 nm | 1.0 | [S41] |

**References**

1. T. D. Kühne, M. Iannuzzi, M. Del Ben, V. V. Rybkin, P. Seewald, F. Stein, T. Laino, R. Z. Khaliullin, O. Schütt, F. Schiffmann, D. Golze, J. Wilhelm, S. Chulkov, M. H. Bani-Hashemian, V. Weber, U. Borstnik, M. Taillefumier, A. S. Jakobovits, A. Lazzaro, H. Pabst, T. Müller, R. Schade, M. Guidon, S. Andermatt, N. Holmberg, G. K. Schenter, A. Hehn, A. Bussy, F. Belleflamme, G. Tabacchi, A. Glöss, M. Lass, I. Bethune, C. J. Mundy, C. Plessl, M. Watkins, J. VandeVondele, M. Krack, J. Hutter, *J. Chem. Phys.* **2020**, 152, 194103.
2. J. VandeVondele, M. Krack, F. Mohamed, M. Parrinello, T. Chassaing, J. Hutter, *Comput. Phys. Commun*. **2005**, 167, 103.
3. a) S. Goedecker, M. Teter, J. Hutter, *Phys. Rev. B* **1996**, 54, 1703; b) C. Hartwigsen, S. Goedecker, J. Hutter, *Phys. Rev. B* **1998**, 58, 3641.
4. J. VandeVondele, J. Hutter, *J. Chem. Phys.* **2007**, 127, 114105.
5. T. C. Lu, F. W. Chen, *J. Comput. Chem*. **2012**, 33, 580.
6. L. Jiao, J. Wu, H. Zhong, Y. Zhang, W. Xu, Y. Wu, Y. Chen, H. Yan, Q. Zhang, W. Gu, L. Gu, S. P. Beckman, L. Huang, C. Zhu, *ACS Catal.* **2020**, 10, 6422.
7. S. Chen, W. Lu, R. Xu, J. Tan, X. Liu, *Carbon* **2023**, 201, 439.
8. Y. Zhu, W. Wang, J. Cheng, Y. Qu, Y. Dai, M. Liu, J. Yu, C. Wang, H. Wang, S. Wang, C. Zhao, Y. Wu, Y. Liu, *Angew. Chem. Int. Ed.*  **2021**, 133, 9566.
9. C. Zhao, C. Xiong, X. Liu, M. Qiao, Z. Li, T. Yuan, J. Wang, Y. Qu, X. Wang, F. Zhou, Q. Xu, S. Wang, M. Chen, W. Wang, Y. Li, T. Yao, Y. Wu, Y. Li, *Chem. Commun.* **2019**, 55, 2285.
10. L. Jiao, W. Xu, Y. Zhang, [Y. Wu](https://xueshu.66557.net/citations?user=btAjtukAAAAJ&hl=zh-CN&newwindow=1&oi=sra), [W. Gu](https://xueshu.66557.net/citations?user=bsr78LIAAAAJ&hl=zh-CN&newwindow=1&oi=sra), X. Ge, B. Chen, [C. Zhu](https://xueshu.66557.net/citations?user=Ousp5IoAAAAJ&hl=zh-CN&newwindow=1&oi=sra), [S. Guo](https://xueshu.66557.net/citations?user=9MRznZ0AAAAJ&hl=zh-CN&newwindow=1&oi=sra), *Nano Today* **2020**, 35, 100971.
11. Z. Lyu, S. Ding, M. Wang, X. Pan, Z. Feng, H. Tian, C. Zhu, D. Du, Y. Lin, *Nano-Micro Lett.* **2021**, 13, 146.
12. Q. Feng, G. Wang, L. Xue, Y. Wang, M. Liu, J. Liu, S. Zhang, W. Hu, *ACS Appl. Nano Mater.* **2023**, 6, 4844.
13. Y. Chen, L. Jiao, H. Yan, W. Xu, Y. Wu, H. Wang, W. Gu, C. Zhu, *Anal. Chem.* **2020**, 92, 13518.
14. G. Song, J.-C. Li, Z. Majid, W. Xu, X. He, Z. Yao, Y. Luo, K. Huang, N. Cheng, *Food Chem.* **2022**, 390, 133127.
15. Y. Wang, A. Cho, G. Jia, X. Cui, J. Shin, I. Nam, K. Noh, B. J. Park, R. Huang, J. W. Han, *Angew. Chem. Int. Ed.* **2023**, 135, e202300119.
16. S. Ji, B. Jiang, H. Hao, Y. Chen, J. Dong, Y. Mao, Z. Zhang, R. Gao, W. Chen, R. Zhang, Q. Liang, H. Li, S. Liu, Y. Wang, Q. Zhang, L. Gu, D. Duan, M. Liang, D. Wang, X. Yan, Y. Li, *Nat. Catal.* **2021**, 4, 407.
17. J. Hao, C. Zhang, C. Feng, Q. Wang, Z.-Y. Liu, Y. Li, J. Mu, E.-C. Yang, Y. Wang, *Chinese Chem. Lett.* **2023**, 34, 107650.
18. S. Ding, Z. Lyu, L. Fang, T. Li, W. Zhu, S. Li, X. Li, J. Li, D. Du, Y. Lin, *Small* **2021**, 17, 2100664.
19. D. Wang, J. Wang, D. Liu, J. He, M. Wang, H. Huang, G. Nie, H. Ding, X. Yan, *Nano Res.* **2024**, 17, 1827.
20. X. Niu, Q. Shi, W. Zhu, D. Liu, H. Tian, S. Fu, N. Cheng, S. Li, J. N. Smith, D. Du, Y. Lin, *Biosens. Bioelectron.* **2019**, 142, 111495.
21. X. Zhu, J. Wu, R. Liu, H. Xiang, W. Zhang, Q. Chang, S. Wang, R. Jiang, F. Zhao, Q. Li, L. Huang, L. Yan, Y. Zhao, *ACS Nano* **2022**, 16, 18849.
22. W. Xu, W. Song, Y. Kang, L. Jiao, Y. Wu, Y. Chen, X. Cai, L. Zheng, W. Gu, C. Zhu, *Anal. Chem.* **2021**, 93, 12758.
23. L. Zhu, H. Zhong, D. Du, T. Li, H. Nguyen, S. P. Beckman, W. Xu, J.-C. Li, N. Cheng, Y. Lin, *Nano Res.* **2023**, 16, 5216.
24. L. Jiao, Y. Kang, Y. Chen, N. Wu, Y. Wu, W. Xu, X. Wei, H. Wang, W. Gu, L. Zheng, W. Song, C. Zhu, *Nano Today* **2021**, 40, 101261.
25. S. Ding, J. A. Barr, Z. Lyu, F. Zhang, M. Wang, P. Tieu, X. Li, M. H. Engelhard, Z. Feng, S. P. Beckman, X. Pan, J. Li, D. Du, Y. Lin, *Adv. Mater.* **2024**, 36, 2209633.
26. Z. Lyu, S. Ding, L. Fang, X. Li, T. Li, M. Xu, X. Pan, W. Zhu, Y. Zhou, D. Du, Y. Lin, *Anal. Chem.* **2023**, 95, 4521.
27. Y. Zhu, W. Wang, P. Gong, Y. Zhao, Y. Pan, J. Zou, R. Ao, J. Wang, H. Cai, H. Huang, M. Yu, H. Wang, L. Lin, X. Chen, Y. Wu, *ACS Nano* **2023**, 17, 3064.
28. W. Wu, S. Xia, Y. Liu, C. Ma, Z. Lyu, M. Zhao, S. Ding, Q. Hu, *Biosens. Bioelectron.* **2023**, 225, 115112.
29. Y. Chen, H. Zou, B. Yan, X. Wu, W. Cao, Y. Qian, L. Zheng, G. Yang, *Adv. Sci.* **2022**, 9, 2103977.
30. N. Feng, Q. Li, Q. Bai, S.C. Xu, J. Shi, B. J. Liu, J. C. Guo, *J. Colloid Interface Sci.* **2022**, 618, 68.
31. Y. B. Li, X. L. Bai, M. M. Xu, S. Y. Xu, G. F. Hu, L. Y. Wang, *Sci. China Mater*. **2016**, 59, 254.
32. Y. Xing, J. D. Xiu, M. Y. Zhou, T. L. Xu, M. Q. Zhang, H. Li, X. Y. Li, X. Du, T. Y. Ma, X .J. Zhang, *ACS Nano* **2023**, 17, 6789.
33. M. Y. Chang, Z. Y. Hou, M. Wang, D. Wen, C. X. Li, Y. H. Liu, Y. L. Zhao, J. Lin, *Angew. Chem. Int. Ed.* **2022**, 134, e202209245.
34. P. Y. Qi, J. Y. Zhang, Z. R. Bao, Y. P. Liao, Z. M. Liu, J. K. Wang, *ACS Appl. Mater. Interfaces* **2022**, 14, 19081.
35. R. Niu, Y. Liu, Y. H. Wang, H. J. Zhang, *Chem. Comm*. **2022**, 58, 7924.
36. Y. D. Zhe, J. Liu, Z. Q. Zhao, Z. Li, K. Li, Y. Q. Lin, *Nanotechnology* **2022**, 33, 505703.
37. P. Huang, J. Lin, W. W. Li, P. F. Rong, Z. Wang, S. J. Wang, X. P. Wang, X. L. Sun, M. Aronova, G. Niu, R. D. Leapman, Z. H. Nie, X. Y. Chen, *Angew. Chem. Int. Ed.* **2013**, 125, 14208.
38. J. Ye, W. B. Lv, C. S. Li, S. Liu, X. Yang, J. W. Zhang, C. Wang, J. T. Xu, G. Q. Jin, B. Li, Y. J. Fu, X. Q. Liang, *Adv. Funct. Mater.* **2022**, 32, 2206157.
39. Z. Y. Yan, X. Wu, W. Tan, J. P. Yan, J. Zhou, S. J. Chen, J. L. Miao, J. Cheng, C. J. Shuai, Y. W. Deng, *Adv. Healthcare Mater.* **2024,** 13, 2304595.
40. M. Y. Chang, Z. Y. Hou, M. Wang, C. Z. Yang, R. F. Wang, F. Li, D. L. Liu, T. L. Peng, C. X. Li, J. Lin, *Angew. Chem. Int. Ed.* **2021**, 60, 12971.
41. L. Wang, X. Z. Qu, Y. X. Zhao, Y. Z. W. Weng, G. I. N. Waterhouse, H. Yan, S. Y. Guan, S. Y. Zhou, *ACS Appl. Mater. Interfaces* **2019**, 11, 35228.
